# Is the ZIKV Congenital Syndrome and Microcephaly Due to Syndemism with Latent Virus Coinfection?

**DOI:** 10.3390/v13040669

**Published:** 2021-04-13

**Authors:** Solène Grayo

**Affiliations:** Institut Pasteur de Guinée, BP 4416 Conakry, Guinea; solene.grayo.ext@pasteur.fr or grayo.solene@orange.fr

**Keywords:** Zika virus, latent virus, coinfection, reactivation, coevolution, tropism, transmission, viral reservoir, persistence, congenital syndrome

## Abstract

The emergence of the Zika virus (ZIKV) mirrors its evolutionary nature and, thus, its ability to grow in diversity or complexity (i.e., related to genome, host response, environment changes, tropism, and pathogenicity), leading to it recently joining the circle of closed congenital pathogens. The causal relation of ZIKV to microcephaly is still a much-debated issue. The identification of outbreak foci being in certain endemic urban areas characterized by a high-density population emphasizes that mixed infections might spearhead the recent appearance of a wide range of diseases that were initially attributed to ZIKV. Globally, such coinfections may have both positive and negative effects on viral replication, tropism, host response, and the viral genome. In other words, the possibility of coinfection may necessitate revisiting what is considered to be known regarding the pathogenesis and epidemiology of ZIKV diseases. ZIKV viral coinfections are already being reported with other arboviruses (e.g., chikungunya virus (CHIKV) and dengue virus (DENV)) as well as congenital pathogens (e.g., human immunodeficiency virus (HIV) and cytomegalovirus (HCMV)). However, descriptions of human latent viruses and their impacts on ZIKV disease outcomes in hosts are currently lacking. This review proposes to select some interesting human latent viruses (i.e., herpes simplex virus 2 (HSV-2), Epstein–Barr virus (EBV), human herpesvirus 6 (HHV-6), human parvovirus B19 (B19V), and human papillomavirus (HPV)), whose virological features and co-exposition with ZIKV may provide evidence of the syndemism process, shedding some light on the emergence of the ZIKV-induced global congenital syndrome in South America.

## 1. Introduction

### 1.1. The Emergence of Congenital ZIKV Syndrome (CZS)

A strange silent period of almost seven years (2007–2015) elapsed between the emergence of the Zika virus (ZIKV) in Oceania, defined by an increased incidence in the classical form of the illness, i.e., “Zika fever” (Yap in 2007: 49 confirmed cases and 39 probable cases), and the South American outbreak (Brazil in 2015: 440,000–1,500,000 reported cases), where the virus marked its re-emergence in powerful and serious forms with atypical clinical manifestations in humans, and shading the French Polynesian outbreak two years before (French Polynesia in 2013: 294 confirmed cases and approximately 8000–19,000 suspected cases) [1,2,3,4,5,6].

As the incidence of neonatal microcephaly increased 20-fold in April/November 2015 compared to the rate in 2010–2014 (CDC, 2017) during the ZIKV outbreak (based on epidemiological studies and reported cases), in-depth investigations are on the rise in an attempt to characterize the detailed features of ZIKV infection in pregnancy and provide evidence of a causal link between maternal ZIKV infection and the recent emergence of specific congenital symptoms [7,8,9,10,11,12]. These data highlight the ability of ZIKV to replicate and disseminate in different fetal organs (e.g., the spleen, lungs, liver, muscle, and brain), supporting the mechanism affecting the embryo and/or fetus development [13,14,15,16,17,18]. The widespread use of various imagery tools, computed tomography (CT), and magnetic resonance imaging (MRI) indicates the importance of non-classical neuroimaging findings (e.g., fetal microcephaly and intracranial calcifications) in Congenital Syndrome (CS) definition(s) [18,19,20,21,22,23,24]. The evidence has been rapidly piling up and strengthens the relationship between ZIKV infection and microcephaly, which has been well summarized in numerous reviews [25,26,27,28]. In light of global public health being in a state of high tension, this evidence has led to the declaration of ZIKV as a new congenital pathogen with potential risk of vertical transmission and association with specific fetal effects (such as microcephaly), referred to as CZS (e.g., in Brazil’s PAHO commitment (Pan American Health Organization), the World Health Organization’s 2015 report (WHO), and the WHO’s 2016 statement). Unfortunately, this sudden attention might result in skews (both quantitative and qualitative) in the form of overestimating the number of ZIKV congenital infections and, also, by attributing an overly strong correlation between ZIKV and observed congenital manifestations influenced by confounding factors [29,30]. By glossing over other alternative determinants, ZIKV is credited as the causal agent of microcephaly as a result of outbreaks and/or its occurrence in endemic areas, while gaps in knowledge still remain [31,32,33,34]. In fact, microcephaly is the most common birth defect-related indicator of suspected congenital ZIKV infection with microcalcifications, which gives rise to two issues. First, no consensus regarding a standard definition of microcephaly has yet been established (i.e., no uniformity exists in its definition) [35,36]. Second, microcephaly is a common feature of several congenital viruses (e.g., human cytomegalovirus (HCMV), herpes simplex virus (HSV), human immunodeficiency virus (HIV), and rubella virus); it could be caused by exposure to toxics (e.g., drugs, alcohol, and other environmental substances and it has already been described in several congenital genetic defects. [37,38,39]). Thus, at first glance, the description of CZS shows no specific findings compared to other etiologies, in particular, congenital viral infections [40].

In the context of its emergence in South America and the impressive phenotype of microcephaly, this can sometimes lead to speculation and, even, to blind acceptance [41,42]. Even if viral RNA isolation in fetal and placental tissues (e.g., brain) may be related to a possible specific tropism regarding ZIKV, this would provide evidence of a correlation, but is not enough to determine whether ZIKV actually causes microcephaly or brain damage [41,42,43,44]. In some cases, the connection of ZIKV to fetal brain abnormalities is established without confirmation via a positive ZIKV test [45], and there is the risk that other arboviruses that co-circulate in similar spatiotemporal contexts are a confounding factor [46,47,48]. In the general thinking of a clinical syndrome as the result of one (single) pathogen isolated from clinical specimens, the detection of other contributing agents is omitted and/or not prioritized in the context of an outbreak [49].

In fact, features of the Brazilian CZS outbreak have become a talking point in relation to the local high concentration of microcephaly cases in northeastern Brazil [50,51,52,53,54]. Campos et al. proposed that “the unusual distribution of microcephaly in the Northeast region might be accentuated by prior, sequential exposition and/or co-viral infection with other pathogen”, suggesting that ZIKV is not the only culprit [55,56,57,58].

### 1.2. Current ZIKV Pathogenesis Image: Mirror of Common Congenital Syndrome?

**Figure 1:**
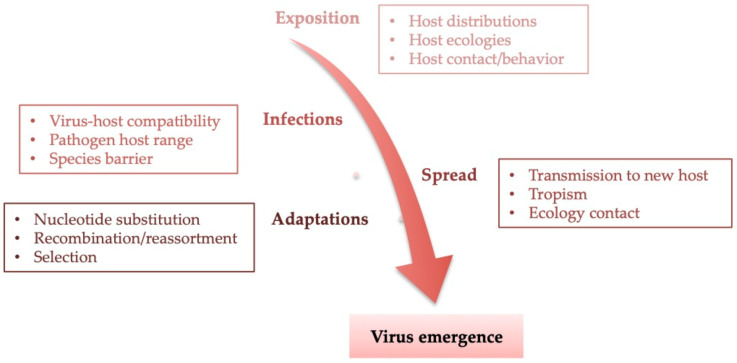
Dynamic process of viral emergence following four steps: exposition, infection, spread, and adaptation. At each step, several biological mechanisms and/or determinants in rectangles can be involved and can result in changes to the viral genome, host tropism, and pathogenicity, providing a baseline for the definition of viral evolution (based on data from Parrish [72], Woolhouse [75], and Ketkar [71]).

Even though the *Aedes aegypti* vector is competent at promoting efficient ZIKV infection of humans in urban areas, viral transmission through human biofluids brings the classification of ZIKV closer to the human pathogens group (1) (human reservoir and “vector”) than to the arboviruses group (e.g., dengue virus (DENV), chikungunya virus (CHIKV), or yellow fever virus (YFV)) [59,60]. The increasing frequency of microcephaly in ZIKV-infected mothers during the early stage of pregnancy reinforces the hypothesis that ZIKV promotes particular tropism (2) in the fetal–placental unit (the neuronal sphere) and that it belongs to the group of congenital pathogens in humans, called the TORCH group (*Toxoplasma gondii*, other pathogens, rubella, cytomegalovirus, herpes simplex virus) [61,62,63,64]. The viral persistence (3) in the female urogenital tract (vaginal secretions, endocervical mucosa, ovary, endometrium, and uterus) means ZIKV is far from having disclosed all of its pathogenesis potential (4) in the reproductive life of women [65,66,67], especially in the context of following particular pregnancy-induced reactivation from genital and/or other ZIKV reservoirs [68,69,70]. The description of CZS from case reports and experimental studies provides a “new image” of contemporary ZIKV with the collapse of the “dead host/reservoir host” boundaries in humans. This view differs from that of the Yap outbreak, which was marked (in the first instance) by ZIKV emergence resulting from a long-term dynamic process occurring in four steps: exposition, infection, spread, and adaptation (Figure 1) [4,71,72,73]. The American outbreak shows the complexity of the ZIKV evolution system following a similar baseline model to the emergence of ZIKV in Yap (Figure 1) by the growing complicated use of determinants. This serves as evidence of a continued risk given ZIKV’s capacity to re-emerge under various environmental conditions originating from new ecological models designed bases on biological mechanism networks [74,75] (Figure 1). In this paper, regarding the phenotypic observation, four specific featural changes are outlined for human infections during the last ZIKV outbreak: (1) transmission mode (ex: vertical transmission), (2) tissue tropism (ex: neuronal sphere)), (3) persistence (ex: genital organ), and/or (4) pathogenesis (ex: teratogenic).

**Figure 2:**
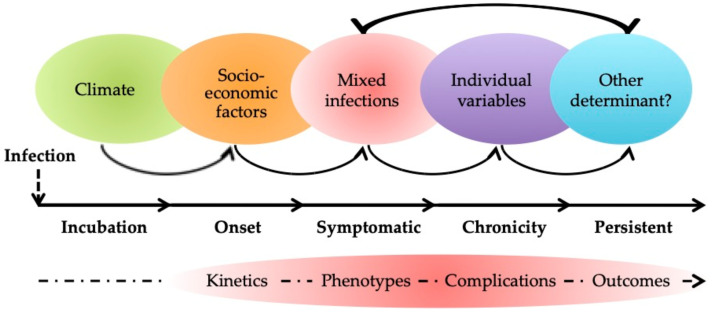
Mixed infections (MIs) influence ZIKV disease in a human host. Once past the incubation phase, ZIKV infection (black straight arrows) may progress along four steps: onset, symptomatic, chronic, and persistent. To simplify, the black straight arrows are here arbitrarily of equal size, but the duration of the steps depends on various conjugated factors. The climate (green circle), socio-economic factors (orange circle), mixed infections (red circle), individual variables (purple circle), and other determinants (unknown) (blue circle) participate in the ZIKV emergence and physiopathology. Each of them interacts with others (round black arrows). The climate promotes the mosquito bites opportunities leading to ZIKV infection in human and the incubation period (first arrow). The climate and the socio-economic factors affect the health status of person and the probability to develop an infection with the onset period (second arrow). The mixed infections (MIs) characteristics (ex: pathogenic composition) depend on the climate and socio-economic factors (round black arrows) and define the ZIKV physio-pathogenicity with the symptomatic period (third arrow). The individual variables, such genetic predisposition, drive the host response, from the clearing infection to the chronic period (fourth arrows). Conjugated with four prior factors (round black arrows), other determinants may make ZIKV persistent in the host (fifth arrows). Depending on composition changes, MIs can partially or totally recast the ZIKV infection characteristics in individual host, influencing the kinetic of the infection, the phenotype, the complications, and the outcome (red oval).

The new potential acquisition may come from singular viral adaptation in hosts as well as interactions with other pathogens that possess these skills [58,76]. The mixed microbial nature of human infections has been recognized since the early 1920s and is heterogenous in global populations [77]. Simultaneous infection of the host by two viruses or more is an important discovery in terms of human health. Although it may well prove to be double-edged, the net results of co-viral infection on human health are mostly disadvantageous (Figure 2) [49]. The clustering of two or more infectious diseases, leading to synergistic or antagonistic relationships that enhance or aggravate diseases or that have negative global health effects on populations living in some environments, is referred to as syndemics (or the syndemism concept) [78,79,80].

Environmental parameters are an important aspect in the emergence of infectious disease outbreaks (Figure 2) [81]. In addition to affecting the life cycle of pathogens and the ecology of vectors, climate also drives socioeconomic factors (e.g., income and education) and environmental management (e.g., building structures, street cleansing, water supply and its storage, and solid waste collection) (Figure 2) [82,83,84]. Certain cases are more susceptible to contracting one or more infectious diseases, which could completely recast the view of the ZIKV system in cases of interactions with other pathogens [85]. ZIKV can learn, be trained, and acquire numerous functions resulting from its contact with other viruses, which can be cooperative, competitive (e.g., interference), or synergistic interactions (e.g., accommodation) [49,86]. ZIKV and other viruses can encounter one another within an organism at different levels and at different periods of ZIKV illness (e.g., phases of incubation, symptomaticity (viremia), chronicity, and/or persistence) (Figure 2), resulting in changes to the classical definition of ZIKV syndrome in terms of kinetics and disease progression, as well as to complications and also phenotype changes that mirror the tissue tropism effects related to ZIKV (Figure 2) [87,88,89].

Prior to defining the nature of co-viral evolution in humans and which benefits provide the co-exposition of ZIKV pathogenicity in pregnancy, the question is: Does the current context meet the conditions set forth for co-viral infection/ZIKV opportunities? Currently, are all conditions set for promoting co-viral infection/ZIKV opportunities? If deemed appropriate, which kinds of coinfections are emerging?

### 1.3. Coinfection Opportunities beyond Arbovirus during the Brazil Outbreak: Human Latent Virus (HLV)

**Figure 3:**
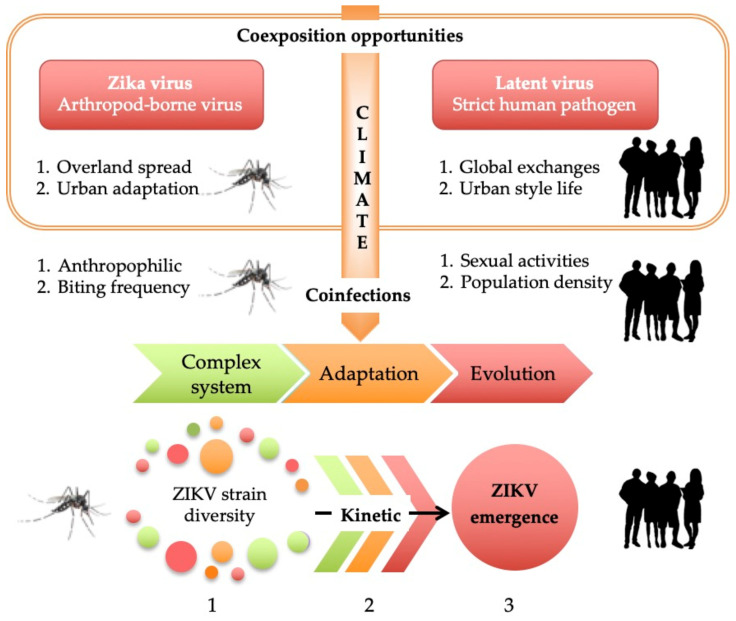
HLV/ZIKV co-exposition to human and co-infection in human to promote ZIKV emergence. Upper panel: two factors promote the HLV/ZIKV co-exposition opportunities to human; (1) the hosts similar distribution (vector and human) due to the global spreading of the mosquito vector (ZIKV host) and the intensification of human exchanges (HLV host); (2) the common preference to urban environment due to the vector adaptation capacity and the human lifestyle change. Middle panel: two factors promote the HLV/ZIKV coinfections in human; (1) the specific hosts (vector and human) behavior with vector anthropophilic and human sexual activities; (2) the contact frequency between hosts (vector and human) through mosquito bites in high density populations. Lower panel: while the climate influences the three biological systems (viruses, vector, and human), HLVs infection may increase the strains diversity of mosquito borne ZIKV in human leading to following sequential process; (1) the adaptation kinetics, (2) leading to ZIKV selection and emergence, (3) with new physio-pathogenicity features.

Despite in-depth research, the mechanism by which ZIKV infection causes microcephaly remains a worrying and mysterious puzzle [90,91,92,93,94,95]. More so than the lack of available in vivo models, ZIKV is a biological complex system whose evolution design depends on the meeting of other biological entities, such as through the occurrence of a mixed viral infection in human. Taken together, this leads to the belief that globalization contributes to the intensification of co-exposition, leading to great opportunities for ZIKV outbreaks (Figure 1 and Figure 3 for ZIKV, latent virus and host) [56,57,58,96]. ZIKV is an arthropod-borne virus, transmitted especially by *Aedes* species. Evidence of other modes of transmission have also been discovered, such as sexual transmission. These two possible modes of transmission result in mosquitos and humans both being drivers of spatiotemporal ZIKV repartition and dissemination [59,60]. The intense co-circulation of arboviruses and their vectors in endemic areas mean that some countries and cities are more involved in coinfection events between ZIKV, arboviruses, and congenital pathogens [39,52,97,98,99,100,101,102]. Areas with particularly dense and heterogenous populations have a higher frequency of and diversity in multiple types of infections (Figure 2 and Figure 3) [103,104]. With the high population density, economic and financial challenges promote the flow of both information and people. These intensive exchanges and activities take place in short periods of time and are concentrated in limited spaces. The differences in urbanism from one region to another, or between continents, may determine the exact epicenter zone of ZIKV appearance, and these determinants differ between Africa and America [105,106]. Today, and with the lack of an emerging infectious disease surveillance system, some regions still seem unaffected by ZIKV re-emergence (i.e., cases are underreported) [87,107,108].

The particular relationship between *Aedes aegypti* and urban humans creates opportunistic environments for both co-viral exposition and coinfection from similar or distinct infection routes (Figure 3) [109,110,111]. The city life promotes opportunities related to human behaviors for ZIKV infection and coinfections via other modes of transmission [59,60,112,113]. For example, the multiple routes of ZIKV infection (e.g., transfusion blood, sexual transmission, and mosquito bites) increases not only the incidence of ZIKV infectious diseases but also the broad occurrence of co-viral infections with arboviruses and sexual and human persistent viruses (Figure 3) [39,52,97,98,99,100,101]. In urban areas, the opportunities for human–human viral infection and to become a carrier of a pool of viruses in their latency period are important from the perspective of intimate contact between two asymptomatic persons [114,115,116].

Humans are a reservoir of the most of persistent viruses resulting from area and/or sex transmission with genital and/or neurotropism and can be reactivated [114,115,116]. In parallel to the behavioral evolution of *Aedes aegypti*, characteristics of human life, such as socioeconomic status and sexual habits, particularly influence the coinfection opportunities in adulthood during the reproductive stage. As a consequence, the abundance and the types of microorganisms within humans change over time and according to the place of residence, and this process continues to shape the immune profile.

As such, it would appear rational to claim that mixed infections are the norm in urban/endemic areas, making some populations more susceptible [117,118]. In contrast, proposing low socioeconomic status as the true contributing factor to greater infectious disease exposition would mask a complex reality with unbalanced access to sanitary care, education, and population behaviors [84,119,120,121]. The urban organization of its habitats is based on socioeconomic determinants, resulting in health problems originating in deprived clusters of social groups. [84,122]. In some areas, certain populations, such as woman and infants, are more vulnerable and are at greater risk of contracting, both early and frequently, latent infection diseases as well as to being exposed to arboviruses, thus mirroring the CZS epicenter area [123,124,125].

Mirroring the syndemism concept, the coinfection arising from initial persistent infection, reactivated by superinfection from another pathogen, such as ZIKV, needs to be investigated, even if it is challenging. In the literature, the “celebrities” HCMV and HIV receive the most attention in relation to congenital persistent viruses in pregnancy [100,126,127,128,129]. Nevertheless, the latent virus group also includes some viruses of the herpes family, such as herpes simplex virus 2 (HSV-2), Epstein–Barr virus (EBV), human herpesvirus 6 (HHV-6), and other pathogens such as human parvovirus B19 (B19V) and human papillomavirus (HPV), which are detected in the female genital tract (FGT) (e.g., the vagina and cervix, endometrial cavity, uterus, and ovaries) (Table 1, Table 2 and Table 3) [39,52,97,98,99,100,101]. All of them affect the FGT and are all public health concerns due the reactivation potential and lytic cycle triggering, which impacts on the reproductive life of women, well-being during childbearing, and oncogenic risks to women’s health (e.g., cervical carcinoma) as well as on pregnancy outcomes and infant susceptibility to later diseases [130]. If sharing similar clinical, biological, and mechanistic features, the “meeting” between ZIKV and a latent virus is a concern for human health.

**Figure A1:**
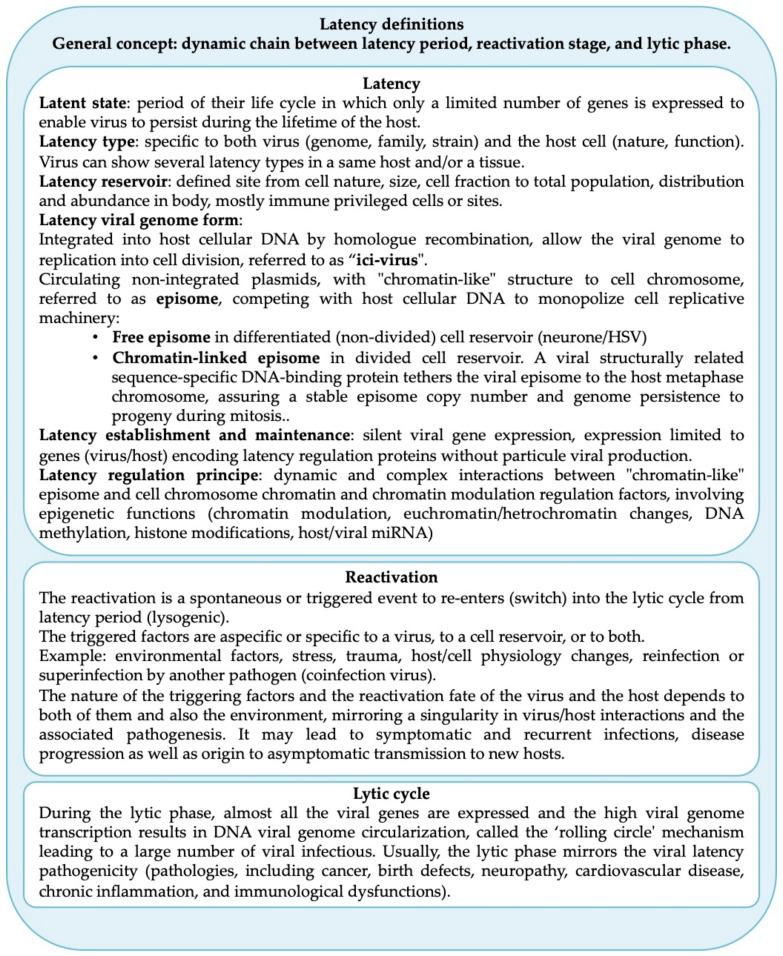
The latency cycle is based on three major steps: latency, reactivation, and the lytic phase. The characterization of viral latency includes the state, type of reservoir, form, establishment, and regulation.

Considering this, the following section reviews the characteristics of the above-listed latent virus biological systems to highlight the key elements of balancing the latency/lytic phase and of triggering the lytic cycle regarding the effects in pregnancy and/or similarities with the ZIKV biological system. In other words, whether common or specific to each virus, latent virus pathogenic mechanisms and their overlap with some of those observed in CZS and others atypical clinical manifestations (Guillain-Barré Syndrome (GBS)) may be evidence of ZIKV’s membership in the latent virus group or, at least, may be an influencing partner (a triggering factor) in the development of later diseases related to latent virus. Figure A1 lists various latent pathogen-related definitions to facilitate reading.

## 2. Human Latent Virus (HLV) Selection with Common Interests in CZS

### 2.1. Herpesviridae: HSV-2, HHV-6, and EBV

**Figure A2:**
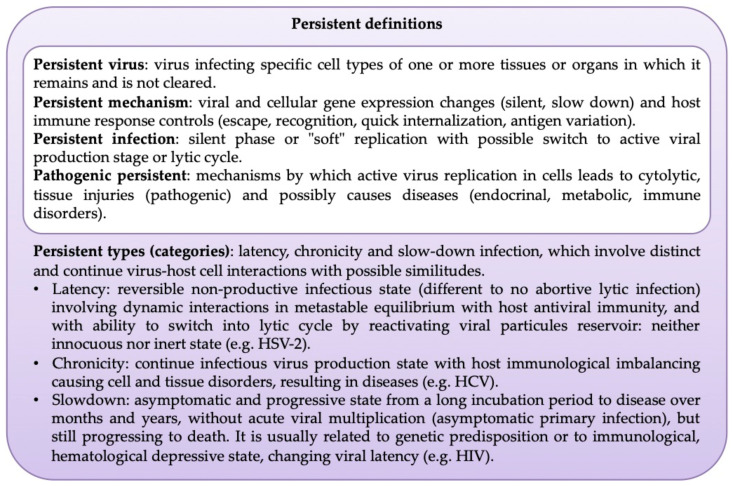
Persistent definitions of intracellular pathogens.

*Herpesviridae* is a family of enveloped viruses with large and linear double-stranded DNA genomes and with three subfamilies, namely, *Alpha-*, *Beta-*, and *Gammaherpesvirinae*. In view of its ubiquity, this group is a global public health concern, affecting human populations at all stages of life (i.e., birth, puberty, reproductive life, and old age) and influencing infant susceptibility in later years following silent in utero transmission [114,131]. The members of the herpes simplex family are transmitted by various biofluids (HLV, Table 1) and persist in the host’s body throughout their life, causing recurrent infections, particularly via latent processes, unlike slow infections and chronic infections (Figure A2).

Each subfamily shows specificities in tissue tropism and in targeted cells as a latent reservoir and/or in performing active replication, which is more or less lytic and characterizes the pathogenicity level (HLV, Table 2). The mechanisms of establishing latency and the reactivation need to be independently discussed, including the trigger factors of reactivation. As a consequence, each subfamily of herpesviruses is examined from one selected referent: HSV-2 (A), HHV-6 (B), and EBV (G) to the *Alpha-*, *Beta-*, and *Gammaherpesvirinae* subfamilies, respectively. Particular attention is paid to their frequent underestimation in pregnancy compared to HCMV and varicella-zoster virus (VZV), and to their causing of congenital syndromes close to CZS.

#### 2.1.1. Herpes Simplex Virus 2 (HSV-2): Female Genital Herpes

HSV-2/1 belongs to the *Alphaherpesvirinae* subfamily, as does VZV. HSV-1/2 replicates with typically short cycles (hours) in epithelial cells and establishes viral latency in sensory neurons [114,132]. HSV-2 herpes genitalis is the most common sexually transmitted infection (STI) [133] (Table 1). The global HSV-2 prevalence is estimated as 11.3%, with a higher rate in Africa (31%) followed by the United States (14%) [134,135]. HSV-2 antibody detection is more frequent in women than men. The level of sexual activity and the number of sexual partners are major risk factors for HSV-2 exposition [136,137,138,139,140]. Primary infection is more often associated with clinical evidence, commonly painful genital lesions. In one study, 24% of patients showed systemic symptoms, such as fever, headaches, and malaise [133]. Typically, 10–14 days post-exposition, alphaherpesvirus latency is established within the cell body’s nucleus of sensory nerves (e.g., cervical, lumbosacral, or autonomic ganglia) [141].

- Latent genome persistence of alphaherpesviruses

HSV-2 adopts a circular form of viral DNA, called the episome. As per non-dividing cells, this episomal plasmid is not attached to the host’s DNA, but still adopts cell chromatin DNA-like structures (Table 2) [114].

- Latent genome expression and maintenance

In HSV latency, transcription is almost completely silenced via mostly epigenetic regulation of modulating chromatin forms, its accessibility, and the expression of chromatin assembly factors from the expression of specific viral proteins that interact with chromatin regulation factors [114,142,143]. The latency-associated transcripts (LATs) code from the long repeat region of the viral genome, which participates in latent infection by antisense regulation to repress expression, promoting repressive chromatin forms (heterochromatin) and showing anti-apoptosis action [114]. The LATs could also be assisted by host- and viral-encoded miRNAs as well as by host DNA methylation in the latency control [114,132,142,143]. Short noncoding RNAs along with long noncoding RNAs (lncRNAs) are LAT transcripts typically involved in the maintenance of latency [114,142,143]. Nevertheless, not all infected latency cells express LATs, and some studies support the function of LATs in reactivation or, at least, in establishing a high risk of spontaneous reactivation [141]. The expression of lytic transcripts in latency is favorable for reactivation events rather than silent states, heightening the susceptibility to triggering factors (Table 2).

- Reactivation

Reactivation of HSV-2, which is resident in sensory neurons that innervate the reproductive tract and dermatome, is related to various types of stress (e.g., clinical interventions, pain, heat, or UV light-induced, emotional changes) and the hormonal treatment of disorders (e.g., glucocorticoid and corticoid) (Table 2) [139,144]. Contrary to past beliefs, the viral reactivation risks of asymptomatic infection are as likely as those that have been clinically evidenced [144]. Recurrent infections may occur all along the reproductive life span; the female mucosal urogenital tract epithelium is more prone to asymptomatic shedding than men’s genital skin [144], especially in pregnancy [138,141]. Pregnancy-induced HSV-2 reactivation provides an opportunity for mother-to-fetus silent transmission, possibly associated with congenital HSV-2 infection (Table 3) [145,146].

- Pregnancy

While in the female urogenital tract the bacterial flora protects against HSV-2-induced vaginosis and inflammation, some findings indicate a reverse effect in pregnancy, with polymicrobial infections benefitting HSV-2 pathogenicity, resulting in fatal fetal outcomes (e.g., spontaneous abortion, low birth weight, and premature delivery) [147]. In response to bacterial lipopolysaccharide (LPS), HSV-2-infected human fetal membrane explants show changes in Tyro3, Axl, and Mer (TAM) receptor expression and in the modulation of innate immune responses (interleukin -1 (IL-1)) [147]. The in vivo model results confirm HSV-2 coinfection-induced inflammation (ILb-1 production) and inflammasome (NLRP3 expression). The HSV-2 reactivation from superinfection by other sexually transmitted pathogens, such as HIV or HPV, may also unbalance the microbiota in the Female Genital Tract (FGT), favoring bacterial vaginosis (BV) and thus enhancing the pathogenicity of HSV-2 [135,148,149,150]. HSV-2 reactivation, in turn, may predispose the FGT to HIV primo-infection by facilitating the inflammatory infiltration of CD4-positive T lymphocyte cells in the FGT [141]. In Rio de Janeiro, Brazil, one investigation showed a high frequency of HHV-2/HIV coinfection in patients with recurrent and primary genital infections [149,151]. The consequences of HSV-2/HIV coinfection and with other viral STI infections are of particular concern in pregnancy. Moreover, a cofactor effect of HSV-2 coinfection in HPV pathogenesis is also reported in experimental and epidemiological studies, associated with a higher risk of developing HPV cervical cancer [148].

- ZIKV/HSV-2 coinfections and pregnancy

Cases of ZIKV/HSV-2 coinfections have already been described in pregnancy in several prospective studies [52]. In addition, one comparative study showed that HSV-2 infection induces cytotoxic and inflammatory effects in the sterile vaginal system, while ZIKV infection alone does not result in obvious epithelial destruction [152].

**Figure 4:**
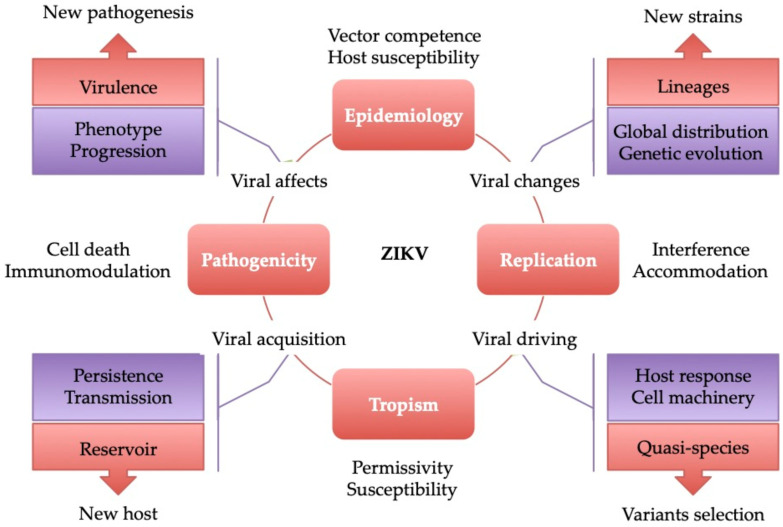
Coinfection with a latent virus influences the ZIKV infection cycle pattern in humans. Replication: In contrast to viral competition (interference), viral transcription is almost completely silenced in latency, benefitting ZIKV in terms of using the cell machinery (purple) for promoting its replication and the selection of new variants (red) without clearing the latent viruses already affecting host response (accommodation). Tropism: Cell specificity in latency establishment offers ZIKV a wide range of reservoirs, changing its tissular tropism (permissive or susceptible), and promoting its persistence as well transmission (purple) to other hosts via new modes (red). Pathogenicity: Coinfections can modulate virus virulence (red) from immunomodulation events and cell death activation (apoptosis), resulting in changes to ZIKV pathogenicity (red), thereby altering disease severity (outcome and phenotype) (purple). Epidemiology: The latent virus may influence ZIKV epidemiology by promoting nucleic acid mutations (substitution) in the viral genome, thus creating new strains (purple), new lineages (red), and/or adapted variant selection, thereby affecting vector competence as well as host susceptibility.

HSV-2 induces immunomodulation and proinflammatory cytokine production (including interleukins IL-8 and IL1β) making it an ideal antagonistic or synergetic partner in enhancing ZIKV pathogenicity in pregnancy (Figure 4: Pathogenesis changes) [152]. ZIKV RNA accumulation in the placenta of pregnant C57BL/6 mice exposed to HSV-2 at day E8.5 (equivalent to the first trimester in humans) and then infected with ZIKV at day E11.5 was significantly higher compared to pregnant mice monoinfected with ZIKV alone (Replication promotion). In HSV-2-induced infection, TAM receptor expression may favor cell susceptibility to ZIKV (entry) as well as permissivity resulting from inflammation (Figure 4: Tropism benefit) [98]. Interestingly, Brazil’s northern region, which has a high number of ZIKV-related microcephaly cases, is also an area with strong seroprevalence for HSV-1 and HSV-2 (Figure 4: Epidemiology influence) [50,149,151,153].

**Figure 5:**
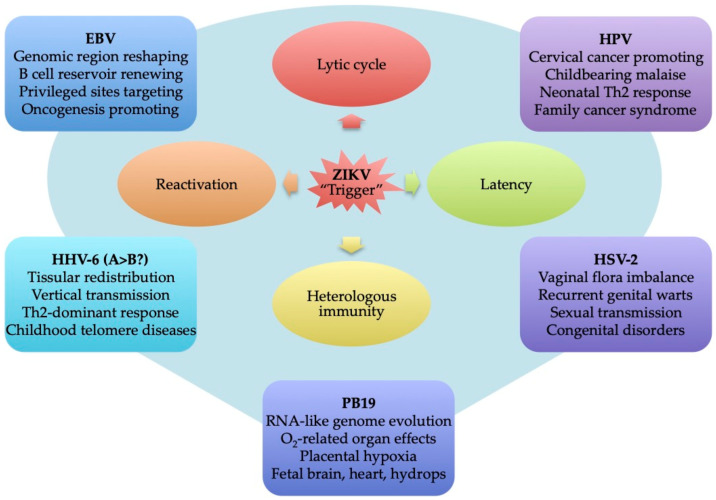
ZIKV infection impacts human latent virus biological systems. ZIKV acts as a triggering factor of reactivation (orange) to promote the switch from latent to lytic replication. In contrast to the latent phase, viral cointeractions in the lytic cycle are mostly competitive events (red) (interference: reduction, blocking, or inhibition replication) to monopolize cell machinery, leading to latent virus exclusion (clearance). ZIKV exposition in the viral latent phase results in cooperation (enhancement and accommodation) or in neutrality (no effect or coexistence), in which interactions are not exclusive (green). Heterologous immunity (yellow): ZIKV coinfection can modify the immune response against latent viruses, providing protective immunity or leading to immunopathology, referred to as the heterologous immune response. Square (blue and purple): depending on latent virus systems (e.g., HSV-2, EBV, HHV-6, B19V, and HPV), mixed infections with ZIKV affect the virus (e.g., genome and pathogenicity) as well as the host (e.g., immunomodulation, tissue tropism, and diseases).

In one study, coinfection with HSV-1 and ZIKV has already been demonstrated in humans with meningoencephalitis [154]. This study suggests an increase in cellular damage associated with ZIKV and the herpes virus family infection and/or in ZIKV facilitating the reactivity of other herpes viruses and neuronal injury by indirect mechanisms (e.g., apoptotic stimulation, inflammasome, and NLRP3 activation) (Figure 5: Reactivation) [154]. Herpes simplex viruses (HSV-1 and HSV-2) share some clinical features and tissue tropism, including of the brain and placenta, with ZIKV, suggesting the possibility of understanding the molecular mechanisms of ZIKV pathogenicity based on HSV findings [39,136,155,156] and/or in a coinfection context (Figure 4 and Figure 5). As suggested above, the influence of HSVs on ZIKV biological systems may concern replication (e.g., a high viral load), tropism (e.g., neuronal and placental tissue targets), and direct or indirect pathogenesis (e.g., apoptosis and immunomodulation), as shown in Figure 4. HSV-1 and HSV-2 may be the partners to promote ZIKV pathogenicity, leading to the emergence of ZIKV-linked GBS and CS in arbovirus endemic areas with high HSV seroprevalence.

Interestingly, a new variant of human HSV-2 from Brazilian HIV-1 coinfected subjects has been identified. Its impacts on the pathogenicity of HSV-2 infection and HIV/HSV-2 coinfection need further investigations [151]. This fact shows the continued evolution of the HSV-2 biological system associated with the coinfection context. In addition, similarly to DENV/CHIKV, HSV-1/2, HPV, and HIV coinfections with ZIKV, multi-complex ZIKV systems need to be considered from more of an epidemiological perspective (e.g., seroprevalence) in order to develop control and prevention strategies (Chapter 3) [157].

- Potential ZIKV/HSV-2 coinfection prevention strategies

To date, few vaccines have been developed against genital HSV-2. From the hypothesis that HSV prevention might limit damage due to ZIKV pathogenesis, efforts to design an effective vaccine against HSV-2 need to be made and, indeed, some works are already in progress [158]. Of particular encouragement is the recent development of broad-spectrum virucidal gold nanoparticles mimicking heparan sulfates that provide nontoxic virucidal action against HSV-2 and ZIKV [159]. Additionally, antiviral activity against HSV-2 is also effective in mice models and ex vivo vaginal tissue culture models. However, continued efforts are required in terms of improving care systems and providing education to reduce inequalities (e.g., spatial, sexual, ethnic, and economic).

#### 2.1.2. Human Herpesvirus Virus (HHV-6A/B): Immunomodulated Viruses

Human herpesviruses 6A and B (HHV-6A and B) belong to *Betaherpesvirinae* subfamily, which includes human cytomegalovirus (HCMV) and human herpesvirus 7 (HHV-7). Compared to the ubiquity of HSVs, HHV-6A/B have a longer (days) replication cycle, and the slower spreading results in characteristic infected cell enlargement [114,160]. HHV-6A/B cause tropism of lymphocyte T CD4+ and can also infect monocytes/macrophages; epithelial, endothelial, and smooth muscle cells; and fibroblasts (Table 1) [161]. The epidemiological, biological, and immunological parameters support differences between HHV-6A and HHV-6B [162]. HHV-6A infection remains uncertain and might be acquired in later ages, showing few clinical signs [163]. However, the presence of HHV-6A DNA in almost half of the endometrial cells of women with idiopathic infertility may support such a cause–effect link [164,165]. By contrast, primary HHV-6B infection typically causes roseola infantum, which occurs in 90% of children within their first two years of life (the sixth disease) [162]. In adults, HHV-6B infection is mostly asymptomatic or responsible for mononucleosis-like syndrome. From here, HHV-6B can enter a state of long-life latency in the lymphoid tissues (e.g., blood, spleen, and lymph nodes) and especially detected within hematopoietic progenitor cells and myeloid cells [166]. In humans, these viruses (HHV-6A/B) persistently infect 70 to 90% of the human population, and the seroprevalence of HHV-6 viruses can be up to 100% in most regions of the world [167]. Several experimental works support that the limited clinical manifestations of singular HHV-6 infection are related to its immunomodulator functions, which promote the establishment of specific viral latency in each immune cell type (e.g., CD4T, CD8, and macrophages/monocytes), providing evidence of a positive coevolution between this virus and humans [168].

- An HHV-6-specific latent form: iciHHV-6

The integration of the HHV-6 genome into host DNA, also referred to as inherited chromosomally integrated HHV-6 (iciHHV-6), corresponds to a unique mechanism of viral latency among herpesviruses. At the terminal regions of its genome, HHV-6 possesses “telomere-like repeats”, allowing its integration near the telomeric junction of host chromosomes by homologous recombination of its direct repeat (DR) region of “TTAGGG” motifs [169,170,171]. The genome is transmitted during cellular division as an integral part of the host genome (Table 2), and from one generation to the other (to 50% of the offspring) via viral integration into germline cells (gametes), i.e., via DNA [172]. Approximately 1% of the human population possesses a copy of HHV-6 in the genome of each somatic cell [173]. Interestingly, iciHHV-6A shows particularly high expression in the brain and testes [174].

- Latent iciHHV-6 regulation

The maintenance of proviral latency is commonly based on restricting viral replication to some latency-associated genes, allowing a limited host immune response and virus persistence in niches until cell death. Transcriptional studies of the iciHHV-6A/B genome describe four latency-associated transcripts of HHV-6A/B (H6LTs), which correspond to ORF99, ORF142, and ORF145 proteins [167]. Expression of the U94 transcript also allows the establishment and maintenance of latency by inhibiting viral lytic replication in HHV-6A/B-infected peripheral blood mononuclear cells. As per herpesvirus latency regulation, RNA-seq analysis from patient-derived iciHHV-6A cells shows various epigenetic process markers to ensure silencing of the HHV-6 virus genome [114]. Moreover, small noncoding RNAs (sncRNAs) such as miR-U86 can prevent lytic cycle activity. By inhibiting the expression of U86 at the transactivation step (initial reactivation), miR-U86 suppresses the shift to the lytic cycle to back to the latency stage [114,167]. Although details regarding latency maintenance are limited and it remains a subject of ongoing investigations, its regulation highlights a possible reversible situation, meaning that iciHHV-6B reactivation can promote replicative as well as non-productive cycles (Table 2).

- Reactivation and related diseases

iciHHV-6B is difficult to isolate, presenting challenges for its study, though a recent study argued that iciHHV-6 is able to reactivate even if superinfection with a second HHV-6B strain occurs (Table 3). This argues not only the ability of iciHHV-6B to reactivate but also the role of the superinfection of other pathogens on the immune state of iciHHV-6-positive patients [167,175]. Some studies have postulated that iciHHV-6 positivity benefits human health by enhancing antibody responses against superinfection by a second pathogen, such as HCMV or EBV [174,176]. By contrast, previous work has indicated that HHV-6A infection promotes EBV and HCMV reactivation, and severe lymphopenia in HCMV-positive patients with high seroprevalence to HHV-6B coinfection are more susceptible to developing serious clinical manifestations [175]. Moreover, the integration of HHV-6A/B in some chromosomes has been reported to potentially generate telomere dysfunctions and/or telomerase mutations with greater risk of developing diseases such as cardiovascular diseases [169,177,178]. The above contradictions need further investigation using cutting edge tools, particularly concerning the reactivation potential of HHV-6B and the impact of the telomere-integrated form on host health. HHV-6 reactivation may also depend on the individual immunological changes occurring all along the reproductive life. However, pregnancy is frequently related to latent virus reactivation, including HHV-6 [179,180,181,182,183].

- Pregnancy

HHV-6B is usually acquired in early childhood (from birth to two years) and explains why cases of primary maternal infection are extremely rare. Moreover, HHV-6B seems unable to replicate in endometrial cells, in contrast to HHV-6A, which could be related to the development of infertility in women [184]. In fact, HHV-6A infection may be especially responsible for the immune profile dysregulation of endometrial cells, and its presence in the vagina represents a risk of sexual transmission to partners as well as vertical transmission to the fetus and to newborns at delivery (at birth) [164,185,186]. Since Hall’s estimation of an HHV-6 vertical transmission rate of approximately 1%, much evidence has accumulated to support this mode of transmission as well as its link to possible congenital impacts [183,187,188]. In utero, HHV-6 is an immunomodulatory factor that promotes cytokine secretion, IL-15/IL-18 in trophoblasts, and a reduction in endometrial human leukocyte antigen G (HLA-G) and Mucine 1 (MUC1) in favor of congenital problems, such as miscarriage. Moreover, HHV-6 stimulates the production of cytotoxic natural killer (NK) cells, which control the level of apoptosis at the decidual surface, but which limit the level of regulatory T cells (Treg) that prevent fetus rejection. By facilitating a Th2 switch environment associated with interleukin production (IL-4, IL-5, and lL-10), HHV-6 also plays a role in infertility, as well as in implantation failure [164]. A recent in vitro study demonstrated that HHV-6A infection dramatically changes the expression profile of miRNAs and control of the trophoblast cell adhesion of endometrial cells and trophoblast cell misattachment on endometrial cells [165]. As per various other environmental factors (e.g., hypoxia, signaling pathways, and epigenetic modification), HHV-6 infection may dramatically affect embryogenesis by dysregulating the production of the more than 500 miRNAs connected to this process.

Although HHV-6 is not considered as important a TORCH pathogen as other betaherpesviruses (e.g., HCMV), all of the above examples provide evidence of the immunomodulatory features of HHV-6A that alter a woman’s reproductive life and, especially, pregnancy outcome. Previous work indicates that HHV-6A infection promotes HCMV reactivation and patients with severe lymphopenia (+) HCMV with a high seroprevalence to HHV-6 coinfection are more susceptible to developing serious clinical manifestations [175].

As a consequence, HHV-6 may trigger its own molecular mechanisms, which alters immune cell interactions at the fetal–maternal interface resulting in fatal fetal outcomes but also influence the immune response to others congenital pathogens in the context of the coinfections in pregnancy.

- ZIKV/HHV-6 coinfection and pregnancy

In view of its high seroprevalence, the frequent detection of HHV-6-IgG (past infection) during diagnosis of ZIKV infection is not surprising [99,189,190,191,192]. HHV-6 and ZIKV infection consequences in the fetal development may be the same with a risk of misdiagnosis from clinical evidence. By contrast, the RNA of HHV-6 has been identified in several post-mortem tissues (e.g., thymus, kidney adrenal gland, and liver) of newborns with CZS. This raises important questions, firstly, about the origin of HHV-6 infection (primo-infection or reactivation) and the subgroup HHV-6A/B. Pregnancy, as well as ZIKV infection, can trigger factors related to HHV-6 replication. As per HCMV, HHV-6 may promote ZIKV infection in utero, mirroring a potential facilitating mechanism of ZIKV tropism and/or local replication. As a consequence, the sequence of virus acquisitions is unclear: iciHHV-6B reactivation or HHV-6B infection may or may not be prior to ZIKV infection in pregnancy. This, more generally, indicates the establishment of a pathogenicity cooperative process between HHV-6 and ZIKV in female urogenital tracts which needs to be further investigated, paying particular interest to the iciHHV-6-positive population (Figure 5) [174]. Population-based studies (e.g., HHV-6), such as of genetic predisposition, may be one future option for developing a better understanding of the ZIKV/microcephaly association.

#### 2.1.3. Epstein–Barr Virus (EBV): Lymphocryptovirus

EBV belongs to the *Gammaherpesvirinae* subfamily, the third subfamily of herpesvirus, which shows both cell- and host-specific tropism. Unlike alpha- and betaherpesviruses, with a classical lytic cycle, EBV infection is characterized by the predominance of a latency period (Table 1) [114]. EBV infects essentially naïve B cells in the blood to promote B cell differentiation and to establish viral latency in memory B cells with a long lifespan (Table 2) [114]. In the same individual, EBV latency can show multiple types of persistence with continuous changes through the adaptation of EBV latency according to the cells’ physiological states (B cells), the origin, and host responses, including their immunological immune state and the environment. EBV is one of the most ubiquitous among the herpesviruses in humans [193]. It is estimated that more than 90% to 95% of adults worldwide present latent EBV infection in their B-lymphocytes. In general, EBV infection is contracted in childhood and is often subclinical. In young adults, it presents more of the classic findings of infectious mononucleosis with viral chronic detection in the saliva and blood [193]. Apart from in the nasal mucosa and tongue, EBV is commonly detected in lymphoid tissues, as well as in the intestines and liver, but with less importance in neural and genitourinary tissues [194].

- Latent genome forms of gammaherpesviruses

Unlike alphaherpesviruses, gammaherpesvirus episomes needs to attach to cellular chromosomes so as to limit the risk of losing the viral genome in the dividing cells, such as B cells. The “tethering” of the viral genomes to cellular chromosomes results from viral proteins interacting with chromatin-binding proteins during mitosis [114,142]. The Epstein–Barr nuclear antigen 1 (EBNA1) protein links specifically to chromatin host factors during mitosis. Origin-binding proteins (OBPs) also contribute to DNA attachment and recruitment of cellular proteins for replication [114,142]. From there, the latent gamma-herpervirus episomes can duplicate in synchrony with the cellular cycle, once per cell cycle and during the S phase (Table 2).

- Common viral latency maintenance strategies

As per HSV, limiting the expression of EBV latency genes (transcription) requires chromatin interactions and changes, implicating epigenetic regulation. The Epstein–Barr nuclear antigens (EBNAs; e.g., EBNA-2 and EBNA-LP) and the latency-associated membrane proteins (LMPs; e.g., LMP1) play a major role in transcriptional regulation of viral silencing through chromatin condensation changes, histone modifications, or at the level of DNA methylation [114,142,143]. The sncRNAs, Epstein-Barr Virus-Encoded RNA 1 (EBER1) and Epstein-Barr Virus-Encoded RNA 2 (EBER2) are also involved in EBV latent cycle regulation. Both cell miRNAs and viral miRNAs drive the maintenance of latency and/or repression of the expression of the viral gene during the lytic cycle, such as in the example of EBV BamHI-A rightward transcript (BART20-5p) miRNA, which targets the EBV genes BZLF1 and BRLF1 [114,142,143]. The viral latency features define each EBV latency type, whereas some of them support the oncogenesis properties via promoting the survival of latently infected B cells (Table 2).

- Latency and oncogenic potential

Direct evidence for the tumor formation potential of the EBV latency period encompasses growth signal induction, apoptosis prevention, antiviral immune control, and genome instability, referred to as the IARC package [114,143,195]. For example, LMP-1 and LIMP-2 promote B cell survival by upregulating NF-κB signaling and mimicking the B cell receptor, respectively. The BamHI fragment H rightward open reading frame 1 (BHRF1) miRNAs and the EBV-encoded small RNAs (i.e., EBER1 and EBER2) inhibit apoptosis and promote passage through the cell cycle [114,143]. Moreover, some mutations can appear and are responsible for triggering the spontaneous lytic phase and, often, increasing cell immortalization and carcinoma risk.

As a consequence of these effects, EBV is a direct causative agent of different forms of lymphoma, such as Burkitt’s lymphoma and Hodgkin’s disease, driven by the factors of nature and the context of reactivation, which leads to oncogenesis [196].

- Reactivation and coinfection

In general, environmental stressors (e.g., chemotherapy and radiotherapy), hypoxia, hormonal dysregulation, and coinfection with other pathogens can induce a switch to the lytic phase. Host immunosuppression by HIV infections is often associated with EBV reactivation, favoring EBV dissemination [197]. A number of studies have been conducted to determine the impact of EBV co-exposition with HIV lymphopathogenic virus on the progression of infectious diseases in terms of kinetics, severity, complications, and chronicity, but especially as a co-trigger of oncogenesis [195]. EBV and HIV promote B cell proliferation from synergic activity, such as through stimulating and binding to B cells by the same cell surface protein complex, such as the programmed death-**1**/programmed death-ligand 1 (PD/PDL-1) pathway [195]. While EBV may promote HIV-1 replication, HIV-1 protein expression growth is advantageous for EBV-driven immortalized B cell proliferation [195]. In other words, HIV infection increases the incidence of each of these B cell malignancies. Following HIV coinfection, EBV induces the expression of LMP1, which mimics CD4+ T cell signals and upregulates NF-κB signaling to promote B cell immortalization, B cell survival and, thus, tumorigenesis (Table 3).

- Pregnancy

The reactivation of EBV may frequently occur in pregnancy [198]. A cohort study estimated a 98% EBV seroprevalence in women and showed that pregnancy status is associated with a 35% EBV reactivation rate [199]. During pregnancy, the transplacental transfer of IgG antibodies from the mother protect the infant against many viruses, including EBV [198]. Although no more proof is provided here for possible EBV reactivation effects on congenital disorders, some studies have shown that, following EBV infection, the placental cells change (i.e., they become larger, longer, rounder, and have less-condensed chromatin and their cytoplasmic volumes are increased) [200,201,202]. Another study demonstrated that EBV alone may infect human syncytiotrophoblast cells associated with interleukin-2 (IL-2) and interleukin-6 (IL-6) secretion [198,203]. It has been shown that EBV is also a perfect partner to (+) HIV mothers [198]. The PD/PDL-1 signaling pathway plays a major role in the maintenance of the placental immunodepressive environment by promoting the secretion of immunosuppressive factors (cytokines), which reduces lymphocyte proliferation in the trophoblasts and amniotic epithelial cells (KIM). For example, the EBV/HIV-induced expression of PD-L1 on antigen-presenting cells results in the inhibition of T cells, making the placenta as an ideal niches for both viruses. Moreover, HIV-1/EBV may also especially perturb the cytokine secretion profile and pro-inflammation cell modulators by favoring a shift from a Th1 to a Th2 response, crucial in maintaining pregnancy [198]. As per the ubiquitous nature of global herpesviruses, their coinfection with HIV is a concern in regard to the outcome of pregnancies in Africa, in which HIV seroprevalence remains high [204]. Each region may be characterized by various co-exposition schemes between EBV and other viruses, specifically abundant in this area, such as ZIKV in the arbovirus region.

- ZIKV/EBV coinfection and pregnancy

In spite of their genetic differences and distinct life cycles, the clinical signs of ZIKV and EBV infections may be confounded, such as for hepatitis, rash, arthritis, encephalitis, and renal diseases. The etiologic importance of ZIKV and EBV to these diseases changes depending on regions and seasons [39,205,206,207], making them difficult to diagnose in arbovirus endemic areas where there is the possibility of co-exposition. Endemic areas provide an opportunity for ZIKV-induced EBV to recurrently reactivate (Figure 5), impacting on ZIKV disease progression (Figure 2) by driving the viruses (EBV and ZIKV) to spread along tissues to new niches according to the immunological and physiological status. The initial regions colonized by EBV can differ, including lymph nodes and B cells, and constantly change. Interestingly, following the route of the respiratory tract, EBV can already be detected in neural and genitourinary tissues [194]. However, no consensus is observed in the literature regarding the risk of ZIKV-induced EBV reactivation. One in vitro investigation showed that fetal membranes (FMs) are fully permissive to herpesvirus monoinfection, such as with HSV-1 and HSV-2, VZV, and HCMV, though with the exception of EBV, HHV-6, -7, and -8 [208]. Curiously, in young pregnant woman from Southern Veracruz, Mexico, fetal post-mortem histological investigations demonstrated codetection of ZIKV RNA with EBV in the cortex and with HHV-6 in the kidneys, as well as ZIKV antigen production in the thymus [99].

The last ZIKV outbreak in South America was associated with an atypical increase in microcephaly in pregnancy which at this point had become a matter of particular concern. In turn, ZIKV-induced EBV reactivation in an immunodepressive context may result in malignancy hyperpathogenicity. Recurrent reactivations lead not only to the renewal of the viral reservoir, but also to a reshaping of the population of EBV strains in genomic regions encoding latency regulation, thus determining the continuous emergence and/or abortion of oncogenic EBV strains. Although rare, the description of one case of placental EBV-associated B cell lymphoma of fetal origin is particularly attention-grabbing [209]. Especially, the increased risk of Castleman’s disease (lymphoma and follicular dendritic cell tumor development) results from EBV infection in pregnancy [210,211]. In arbovirus endemic areas, co-exposition of EBV/ZIKV may result in the development of a pathogenic cooperative mechanism to improve the adaptation of each virus in humans, thus enhancing the risk of oncogenesis in the mother and during fetal development in pregnancy (Figure 4 and Figure 5).

- Potential ZIKV/EBV coinfection mechanisms

In view of HIV co-exposition experiences, EBV may have an immunomodulatory effect on ZIKV protection from the female urogenital tract response by (1) reducing the percentage of B cell and T cell pools in the female genital tract; (2) causing imbalance in Th1/Th2 as a switch necessary for placental maturation; and (3) a disruption of IFN–JAK and pro-inflammation signaling of the trophoblast layers. EBV control over the production and secretion of cytokines (IL-2 (EBI3/p355 subunit), IL-6, and IL-12) [212,213,214,215] and the production of IFN gamma defines the placental permissivity to ZIKV and its effects on barrier integrity. Even though not yet clearly demonstrated, PD-L1, which expression is modulated by EBV and HIV, may be involved in CZS. Finally, the anti-apoptotic action of EBV via EBV-BHRF1d/BNIP3 proteins may contribute to ZIKV placental replication and dissemination to particular epithelial cells, such as Hofbauer cells [216,217].

### 2.2. Other Latent Virus Concerns: Human Parvovirus and Human Papilloma Virus

#### 2.2.1. Human Parvovirus B19 (B19 Virus or B19V)

Human parvovirus B19 (B19 virus) is a small single-stranded DNA virus belonging to the *Parvoviridae* family. B19 is the only member of the genus *Erythroparvovirus*, which reflects its restricted replication to erythrocyte precursors (e.g., bone marrow and fetal liver) (Table 1) [218,219]. B19 virus is generally contracted in early childhood and is responsible for the most common rashes in school-aged children, such as slapped cheek syndrome or fifth disease (behind measles, scarlet fever, rubella, and Duke’s disease) [220,221]. Once the acute phase is over, B19V may persist in various human sites, such as the bone marrow, synovium, liver, spleen, and testes [218], mirroring lifelong viral niches as well as further emerging patterns of human pathogenesis [220]. Although the diseases are mostly autoresolved, B19 virus infection is also linked to hepatitis, cardiomyopathies, respiratory illness, autoimmune diseases, and neurological disorders (e.g., encephalitis, encephalopathy, and meningoencephalitis), and is often associated with the host hematological and/or immunological state [220,222].

- From latency to the immunogenic process

In contrast to herpesviruses, the research on the persistent features and nature of B19 virus (e.g., latent, chronic, and slow down infection) is in the early stages (Table 2). Comparative studies with the adeno-associated virus (AAV), a human *Dependoparvovirus* belonging to the same family as B19V, support that B19 virus might mimic the AAV process to establish stable latency by integrating into the human chromosome in a site-specific manner, referred to as the proviral form [223]. In details, B19V shows identical terminal repeat sequences to AAV (i.e., palindromic repeat sequences), serving in the packaging of DNA and integration [223]. Due to this structural homology, P19 may adopt a proviral form in which the virus acts as an integral part of the host genome. Nevertheless, direct evidence of B19 virus latency integrated forms is lacking.

- From latency maintenance to the lytic process

Downregulation of the B19-induced cell DNA replication in erythroid cells is the hallmark of B19V survival in humans. In fact, B19V takes advantage of host cell division to preserve its genomic stability and integrity by inducing a DNA Damage Response (DDR). This B19-induced “DDR” activation leads to cell division and arrest in which the virus hijacks the cellular DNA replication machinery for its own benefit, which consequently affects host genome reparation and the resumption of cell cycle progression [218,223,224]. Transcriptomic investigations of B19-infected human erythroid progenitors (EPCs) confirm changes in cellular gene expression leading to global downregulation of cellular DNA replication, while viral DNA genome replication takes precedence. Instead of participating in the host DNA repair processes, the host DNA polymerase and available DNA replication factors are monopolized to the initiation of the viral lytic phase [224]. The destruction of red blood cell precursors and erythropoiesis blockage are a key pattern of B19 virus lytic activity with oxygen-triggered factors [225]. This hypoxia also contributes to B19 activation by upregulating erythropoietin (EPO) and its receptors (EPOrcp) via promoting JAK2 and STAT5, ERK, and PI3 kinase [218,224,225]. These viral non-structural proteins (NS proteins) mainly play an important role in successful viral multiplication. The NS1 protein upregulates the IL2 inflammatory response, STAT3/PIAS3 signaling pathway, inflammation via NF-kappa beta, and IL-6 expression [218,224]. It remains uncertain as to whether the persistence of B19 virus in humans is correlated with productive or unproductive infections and whether there is any cell specificity, e.g., for erythroid versus non-erythroid cells. To date, the placental endothelial cells are the only non-erythroid cells in which the B19 virus replicates [218]. B19 virus infection in the mother may create a particular pathogenicity outcome, leading to severe congenital complications; in particular, microcephaly is a feature common of cellular “DDR” disorders in ZIKV congenital infection [226].

- B19V particular congenital context: reactivation and/or vulnerability or hazard

Although rare, B19V vertical transmission can still occur without clinical evidence in mothers [227]. B19 vertical transmission increases with pregnancy progression, namely, 15%, 50%, and 60% in the first, second, and third trimesters, respectively, and is linked to a growing demand of the fetus from the mother in terms of energy and oxygen from erythrocytes and EPO regulation erythropoiesis [228,229]. The pregnant weeks of 13 to 20 weeks correspond to the establishment of a connection between with maternal blood and fetal circulation, which is temporarily subjected to hypoxia events and inflammation. Hypoxia-induced inflammatory foci result in a complicated, maternally mediated cellular immune response at the placental interface, while the barrier permeability increases and, as a result, facilitates B19V accessibility to the fetal erythroid progenitor cells associated with an immature immune system [230,231].

The destruction of these red blood cell precursors in the fetal liver by conjugating cytotoxic apoptosis and cell lysis is the key pattern of the B19V feto-pathogenicity mechanism [232]. Anemia, hydrops fetalis, and cerebral artery blood flow increases are consequences of the blocking of fetal erythropoiesis [227]. B19V-induced neuronal disorders and cardiac failure in fetal development are evidence of one oxygen-dependent pathogenicity mechanism [222,227]. In addition, the neuronal cells and fetal cardiomyocytes express the globoside protein. Globoside (i.e., P-antigen or Gb4) and its co-receptor, α5β1 integrin, are described as the primary major receptors for B19 infection [233,234] and are highly expressed in human erythroid cells. Globoside is detected on villous syncytiotrophoblasts and extravillous and villous cytotrophoblasts of the placental tissue [229,235]. Moreover, B19V-NS1 and B19V-VP2 viral proteins increase the expression levels of globoside and its co-receptor, β1 integrin, in BeWo cell cultures, which could facilitate B19V transmission at the maternal–fetal interface [229,236]. Nevertheless, studies have proposed that rather than promoting viral cell internalization as a B19V receptor, globoside may be only involved in the post-entry of viral replication [229,237,238]. To date, evidence of B19V infection as the direct cause of congenital disorders is still lacking [239]. The pattern of B19V infection in pregnancy may be rather associated to a boost in the pathogenicity of other coexposed pathogens, causing severe clinical complications [220,223]. For example, interaction of the HIV gp120 protein with the globoside receptor is a feature shared by B19V, supporting the B19V/HIV cooperative concept and also possible HIV-induced B19V reactivation [240,241,242].

- Arbovirus/B19V coinfection

In Brazil, B19V is the most frequent cause of rash/fever infections in cases where serum testing is negative for arboviruses and rubella, and several studies have shown an increase in PB19 rash/fever cases in a spatiotemporal manner compared to arboviruses and rubella [243,244]. Parvovirus B19 has been detected in 17% of cases when assaying the plasma of Brazilian patients with dengue fever-like symptoms, while samples were negative for arbovirus and were originally misdiagnosed (falsely positive for DENV) [245,246,247,248,249], A similar trend has also been reported for ZIKV [52,250,251].

Infections due to B19V and ZIKV pathogens share similar clinical manifestations, suggesting an overlap of some pathogenicity mechanisms [39,40,248,252,253,254]. The B19V genome is characterized by high rates of observed nucleotide substitutions (1–3 × 10^−4^ substitutions/site/year), which are comparable to the substitution rates of ssRNA viruses that mostly correspond to the groups of re-emerging viruses, including ZIKV. The natural evolution of the genomes of these viruses might contribute to extending, and thus continuing, the spectrum of possible host cells, mirroring a long-term coevolution with humans but also, potentially, its pathogenicity changes (Figure 4) [255,256,257]. These opportunities to set up viral (B19V/ZIKV) coevolutions that facilitate the adaptation to ZIKV in humans, growing in genetic diversity and the development of new atypical types of pathogenicity, are of particular concern in terms of the outbreak context and vulnerable populations such as mothers and infants [258].

- ZIKV/B19V coinfection in pregnancy

In South America, there is a conflicting picture regarding whether the increasing incidence of microcephaly mirrors the emergence of Congenital ZIKV Syndrome in the context of an outbreak [27]. The recent ZIKV/B19V codetection in fetal tissue lesions of a severe congenital case with hydrops fetalis casts doubt over the willingness to assign responsibility to ZIKV for all fetal pathology in areas of high arbovirus density [101,259]. In fact, both of these viruses target the fetal–placental unit associated with singular pathogenicity mechanisms, which can combine to create new congenital phenotypes, offering even more possibilities for fatal effects in the fetus and mother [260,261]. In pregnancy, the B19V-induced erythropoiesis dysregulation affects fetal development by hypoxia-induced brain damage disorders and DDR promotion, resulting to hydrops fetalis. In arbovirus endemic areas, B19V could act by promoting Congenital ZIKV Syndrome. Thus, the increasing incidence of B19V in some areas of Brazil deserves more attention due to the possibility of ZIKV/B19V syndemism.

#### 2.2.2. Human Papillomavirus (HPV): Human Cervical Cancer

Human papillomavirus (HPV), a non-enveloped, double-stranded, circular DNA virus, belongs to the *Papillomaviridae* family with currently almost 200 listed HPV subtypes (Table 1) [262]. HPV infects only humans, both males and females, and can replicate in the skin and the mucosa epithelia of the mouth, throat, and urogenital tract as well as their associated secretions [263,264]. HPV can be contracted by direct skin contact or by other forms of intimate contact (e.g., vaginal and anal sex) [264,265]. HPV is, globally, a major sexually transmitted infection and occurs at least once in a person’s lifetime [262,263]. The major risk factors for acquiring additional HPV subtypes include multiple sexual partners, smoking, using immunosuppressants, and pregnancy [262]. Several types are carcinogenic and classified in low- or high-risk categories. For the low-risk HPV types (LR-HPV), the majority of clinical lesions present as warts rather than as malignancies [263,266]. HPV types 6/11 are responsible for condyloma acuminatum in the urogenital tract (genital warts) as well as juvenile and adult recurrent respiratory papillomatosis [262,263]. High-risk HPV subtypes (HR-HPV), in comparison, are responsible for high-grade intraepithelial lesions in male (i.e., the penis) and female (i.e., the vulva, vagina, and cervix) urogenital tracts which progress into malignancies. All cervical cancer cases are due to HPV, and the majority of cases is caused by the HPV-16/18 subtypes. HPV is responsible for all cases of cervical cancer (50% due to HPV-16) and 70% of vaginal cancer, with HPV-16 being the causative subtype 50% of the time [263]. Although most high-risk HPV infections are resolved in less than two years, the ability of some to persist throughout the lifetime increases the risk of developing HPV-associated cancers [267].

- Latent forms and maintenance

At least two HPV DNA latency mechanisms have been proposed, with possible overlap, but are especially related to cancerogenesis. Oncogenesis as related to specific HPV subtypes may result from the latency process linked to particular strains, according to episomal versus proviral forms (Table 2).

Restricted to the epithelium basal cells, the establishment of HPV DNA in the episomal latent form is associated with expressing a limited number of key viral proteins which, together, establish and maintain HPV latency (e.g., E2 E6, and E7 viral genes) [264]. To support viral DNA replication and transcription, HPV uses host DNA replication mechanisms which induce DNA damage responses (DDRs) and host genomic instability. Various epigenetic processes are involved in facilitating chromatin access, interactions with associated factors, and especially viral genome tethering to chromosomes via the HPV E2 protein (e.g., histone modulation and chromatin condensation level changes) [267]. During cell division, the HPV E2 protein links with the host cell chromatin through the hypermethylation of its specific binding sites and, thus, promotes transcription of the viral E6/E7 proteins [267]. The E6 and E7 tumor activators, by repressing the p53 and pRb proteins, respectively, override the classical cell cycle pattern to “S phase quiescent”. While E6/E7 promote neoplastic transformation of the targeted tissue, E2 regulates viral replication in non-cycling cells, resulting in tissue hypertrophy [267]. The E5/E6/E7 protein complex is also a suppressive immune factor associated with the downregulation of the transcription of interferons (IFNs), cytokines, and IFN-stimulated genes (ISGs), as well as the inhibition of the JAK–STAT signaling pathway, making it a perfect environment for virus reactivation.

However, from recent evidence regarding viral recombination with chromosomal DNA, HPV16/18 can persist in proviral forms. Integration into the host’s DNA genome may concern cancer-derived epithelial cells more, in which the increase in the integration of viral DNA and its methylation is correlated with disease progression [268]. In addition, HPV DNA host integration at fragile sites, which are more susceptible to strand breakage and chromosomal aberrations and instabilities, may favor the epithelial oncogenic process due to stress or viral reactivation [269]. Apart from toxic exposition (UV light, smoke), the risk of triggering HPV reactivation is also associated with pregnancy (Table 3).

- Pregnancy

The persistence of genital warts from past HPV infections appears to be associated with a high risk in women for progressing to cervical cancer over several decades [264,270]. In pregnancy, HPV-16 reactivation increases the risk of tumor development during pregnancy, such as through hydatidiform moles. Pregnancy-induced HPV reactivation is one way in which the newborn can become infected with HPV without involving reinfection of the mother, including through possible sexual transmission from a partner [271,272]. The HPV vertical transmission mode from the mother to the infant could be a result of various propositions, i.e., periconceptual (fertilization), prenatal (transplacental hematogenous transmission or ascending infection), or perinatal (delivery) [273,274,275]. HPV transmission rates are estimated to be between 18.2% and 53.3% for all trimesters [271,272]. Indeed, HPV-16 DNA in syncytiotrophoblast cells was detected in 29% of the samples collected from a spontaneous abortion group [264]. During pregnancy, HPV16 infection promotes Th2 cytokine dominance at the fetal–placental interface by stimulating IL-5, IL-10, and IL-17A expression, which extends to the newborn’s system, persisting after birth for at least several months [271,276]. As this persistence in the newborn’s system can last for at least several months after birth, the HPV16-induced Th2 profile may impact on the postpartum HPV-specific immune response to a subsequent HPV encounter, may influence latent form establishment, and may predispose children to developing HPV lymphoma at a later age [271,272,277]. In addition, this phenomenon might mirror the familial tendency to develop HPV-related cancers. This HPV-induced Th2 profile of prenatal memorization/prolongation is opposed antiviral Th1 maturation, increasing the vulnerability for others genital infections [271,272]. In turn, pathogens responsible for surrounding genital warts (i.e., HSV-2) are potential triggers for HPV reactivation. EBV, HBV, HCV, HSV-2, and B19V are detected in HPV-positive tumors [148]. Although not being significantly associated with a cancerogenesis cervix, the seropositivity against these multiple infections increases the relative risk of developing HPV-16 cervical cancer [148,278]. Several epidemiological and experimental studies support the idea of the mechanisms by which viral cofactors might contribute to HPV tumorigenesis (Guidry’s review offers a well-detailed account), namely EBV-enhanced HPV genomic instability [148], HSV-2 “hit-and-run” HPV oncogenesis [148,279,280], and HHV-6-limited HPV clearance [278]. The cooperation depends on the endemic area. In South America, the higher risk of HIV/HPV coinfection leading to cervical cancer is a particular concern because of its young population [281]. Epidemiological studies have published a prevalence rate of 28.4% for high-risk types of HPV infection (HR-HPV) in (+) HIV Brazilian women [282]. Another investigation in the south of Brazil confirmed the high prevalence of HIV–HPV coinfection (40,8%) compared to HCV (10.8%) and HBV (2.3%). Furthermore, high-grade intraepithelial lesions or in situ carcinoma were found in 52 (9%) of the coinfected cases [281]. Moreover, in a Brazilian (+) HIV pregnant study, the presence of high-risk types (HPV-16/18) in 79.8% of samples with multiple infections in 16.3% of cases was discovered [283].

- ZIKV/HPV coinfection and pregnancy

During the ZIKV outbreak in Guayaquil, Ecuador, the same number of positive results between ZIKV (+5) and HPV (+5) (“pap smear”) with RNA cervical detection were found in 19 samples of atypical squamous cells of undetermined significance (ASCUS) [97]. Even in the absence of a coinfection context here, this prime investigation highlights the need to be aware of (1) the possible merge in etiology between HPV and ZIKV, (2) the risks of ZIKV-induced reactivation in mothers with HPV genital warts leading to cancerogenesis, and (3) the uncertain pregnancy outcomes in an HPV/ZIKV co-exposition context, especially when there are asymptomatic (mother) patients and/or considering the HPV latent form [97,284,285]. An HPV-infected mother may change her placental resistance immune profile to promote ZIKV crossing earlier, for longer, and in greater amounts, leading to in utero fetal damage or CZS. ZIKV, in turn, could be a triggering cofactor for HPV oncogenesis, which dramatically affects well-being during childbearing as well as making infants more susceptible to developing non-communicable diseases and tumors later in life, even in the absence of evidence of direct viral transmission [148]. From one cohort study, the results demonstrated that, rather than being detected at birth, manifestations of CZS may appear during the first year of life [57,286].

The overlap between high HPV seroprevalence groups and the area of CZS emergence is far from indicative of a problem in South America and in particular population clusters [287,288,289,290]. This encourages research to fill the current gaps in literature concerning the epidemiology of the frequency of ZIKV/HPV co-exposition by gender, localization, and other socioeconomic factors to uncover evidence of possible HPV/ZIKV syndemism. The association between the high prevalence of oncogenic HPV and high-risk strains might be verified and/or completely revisited from arguments for the coinfection influence in the HPV carcinogenesis.

Studies on the influence of co-viral infections on ZIKV epidemiology and pathogenicity, as well as on human immunity and health, are ongoing. To date, as direct evidence is lacking, the proposed contribution of HLV coinfections to ZIKV outcomes is only based on speculation. However, this section shows that some HLV biological system features may also overlap with those of ZIKV and influence its viral replication, tropism, pathogenicity, and epidemiology parameters (Figure 4). Therefore, the reciprocal is also true as following describing (Figure 5).

## 3. The Influences of ZIKV Infection on Human Latent Virus (HLV) Outcomes in the Host

Human latent virus refers to one category of persistent pathogens in humans (Figure A1 and Figure A2), in which the maintenance and control mechanisms may be completely changed in the context of a recent ZIKV infection with consequences on latent virus and host reservoirs. Figure 5 argues on this. The followed sections (from Section 3.1, Section 3.2, Section 3.3, Section 3.4 and Section 3.5) support and provide additional explanations to Figure 5.

### 3.1. ZIKV and HSV-2

**Figure 6:**
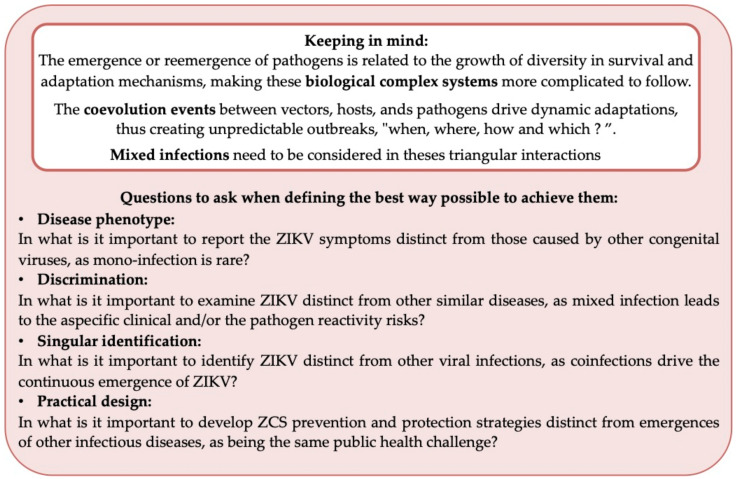
Important considerations for future latency-related CZS research investigations.

ZIKV may play an important role in the HSV outcome by unbalancing the vaginal flora and by promoting recurrent genital warts, sexual transmission, and HSV-related congenital infection (Figure 5). To date, little evidence has been provided regarding ZIKV-induced reactivation of latent viruses, apart from a case of encephalitis in a Brazilian child with ZIKV infection that was related to VZV reactivation in the central nervous system [291]. This lack of data needs to be addressed by designing experimental models such as ZIKV/HSVs coinfection systems that allow the molecular mechanisms to be described and to investigate some potential cooperative signaling pathways associated with changes in tropism as well as in transmission and in the congenital and neurological pathogenicity of both viruses (ZIKV and HSV-1/2) (Figure 5 and Figure 6) [292]. ‘Combination (two-in-one) therapy’ is still considered an appropriate and encouraging technique, as mentioned in Section 2.

### 3.2. ZIKV and HHV-6

In contrast to HSV-2, HHV-6 shows ubiquitous distribution in humans, suggesting a large diversity in susceptible human cells. Therefore, ZIKV infection may drive HHV-6 concentrations in particular compartments, such as immune privileged organs (e.g., the testes and brain). To date, the consequences of HHV-6 infection on human health (e.g., diseases and comorbidities) remain underexplored, including in vulnerable populations. In pregnant woman ((+) HHV-6A), ZIKV infection could facilitate HHV-6 vertical transmission by reducing the barrier integrity (e.g., placental injuries) and contribute to maintenance of the postnatal neonatal immunodepressive mechanisms by promoting HHV-6-linked Th2 cytokine dominance. In addition, ZIKV can promote the emergence of non-communicable diseases (NCDs) and HHV-6-related infant diseases at later ages (e.g., in telomere diseases). Overall, this encourages the design of future studies that include population genetics as a (causal) factor in ZIKV-linked microcephaly. A particular interest in HHV-6(+) clusters coinfected with ZIKV and, ideally, in pregnant woman may potentially show changes in causal links. ZIKV could be victim to false accusations and may only be partially responsible for HHV-6 reactivity, the true responsible party for congenital effects.

### 3.3. ZIKV and EBV

EBV is ubiquitous in human populations, which makes its coinfection with other pathogens common and not unusual with ZIKV (Chapter 2). EBV latency is characterized by a multiplicity of persistent types in (Figure A2). This diversity may originate from the various cointeractions with different viruses, which involve coevolution mechanisms in some niches. As per other pathogens coinfected with EBV, ZIKV represents a determinant of EBV reactivation. It can also control the EBV cellular invasion level, the infected B cell pool, and their relocalization. However, to date, no common pathway has been elucidated. Although being a sufficient factor to cause oncogenesis, ZIKV may exert pressure on the selection of some variants, mirroring a growth in the adaptation and possible oncogenesis of EBV depending on the host. Pregnancy is a particularly vulnerable period in which EBV/ZIKV coinfections show dual risks in terms of the outcome of the pregnancy (fetal development) as well as in terms of the mother’s health in relation to EBV tumorigenesis. While EBV/ZIKV coinfection may affect well-being during childbearing, it also raises questions about the consequences of asymptomatic forms and how to investigate such scenarios.

### 3.4. ZIKV and B19V

Despite differences in the nature of its genome (e.g., DNA vs. RNA), B19V shows as strong a susceptibility as ZIKV to mutation. As such, ZIKV coinfection is an opportunity for B19V viral genome evolution, resulting in growth in its adaptation to the host and possibly in virulence. B19 pathogenicity is related to oxygen-dependent organs such as the placenta and brain. Compared to HHV-6, B19V is closer to TORCH groups, including ZIKV. Pregnancy and ZIKV are both triggering factors for B19 reactivation. As placental development and fetal growth are particularly oxygen-demanding, the second trimester is a vulnerable period for B19V infection, in which ZIKV may dramatically exacerbate congenital effects (e.g., placental injuries and hydrops), embryopathies, abortion, and stillbirth. In view of the seroprevalence of B19V increasing in the latest ZIKV outbreak in Brazil, a full review of some of the studies on ZIKV pathogenicity in B19V endemic regions might lead us to reconsider B19V as a direct cause of congenital effects rather than a cofactor of ZIKV.

### 3.5. ZIKV and HPV

HPV infection in humans shows an evolution closely related to the female urogenital tract compared to the other latent viruses reviewed here. Despite being defined as a latent virus, HPV reactivation from superinfection, such as with ZIKV, may dramatically affect the reproductive life of a woman. While some subtypes (e.g., HPV-16/18) are always responsible for all cases of cervical cancer, ZIKV coinfection with HPV can promote the malignancy of other subtypes (expanding the panel of malignant HPVs) as well as the frequency of tumorigenesis by creating a stress environment. By acting as a trigger, ZIKV may, at the same time, highlight a familial predisposition of HPV-linked cancers which may help in better understanding and management of them, similar to B19V or HHV-6 above. Currently recommended in young adulthood, the consequences of the HPV vaccine prior to ZIKV infection have to be investigated regarding the risks of vaccine inefficacy, particularly in uncontrolled HPV reactivation.

At this stage of review, the contribution of HLV to the re-emergence of ZIKV with congenital syndrome development could result from (1) the transmission of HLV function to ZIKV, improving its outcome in humans and its dominance in the host biological systems, and/or (2) the establishment of cooperation or confrontation systems, even temporarily (partially), suggesting the persistent benefits of ZIKV to HLVs. As described here, ZIKV interactions with HLV may correspond to triggering events (reactivation) and also to altering (interference) or creating benefits (accommodation) for the replication of both. The resulting heterologous immunity of mixed infections is mostly negative for human health (pathogenicity), but the possibility of the opposite effect (positive) needs to be kept in mind (i.e., ZIKV coinfection benefit in host microbiota health) (Figure 5).

The coevolution between viruses and hosts prompts consideration regarding the inclusion of additional variables, including the field. As mentioned in Chapter 1, several other factors influence or drive the cooperation and/or competition of evolution in humans, including the environment, socioeconomic status, host, and lifestyle (Figure 3, Figure 4 and Figure 5). The latter can provide clues for mapping, describing, and classifying the opportunities of coinfection. These take an important place as decision-making tools regarding the future challenges of coinfections or, more broadly, mixed infections with endemic area biodiversity and vulnerable populations (e.g., mothers and infants).

## 4. Update of the ZIKV Congenital Infection Technical and Control Guidance in the HLV Coinfection Context

This title led us to revisit the ZIKV Action Plan: (1) exhaustive and powerful diagnostics, (2) suitable mother and infant management, (3) ZIKV vaccination, (4) redesign of other preventive plans, (6) environmental surveillance with respect to biodiversity, and (7) the development of epidemiological and experimental models. As an introduction, Figure 6 proposes (or lists) some opening questions to support the next useful investigations into CZS (Figure 6).

### 4.1. Perspectives for Diagnoses

Despite efforts to perform screening surveys to define a seroconversion range (between 1% and 50% in the population) for potential vaccine implementation, the seroprevalence of ZIKV infection in endemic countries remains unknown, with even less known regarding ZIKV coinfections. In addition, the search for secondary causes or the detection of other pathogens contributing to the clinical manifestations and disease progression has been far from systematic, in particular, with regard to an outbreak situation.

In fact, this state of mind is reflected in the current availability of diagnostic assays showing high specificity and, thus, glossing over the relevant supplemental pathogens. The matter of coinfection in arbovirus diagnosis, including ZIKV, is already a subject of discussion, with the risk of misdiagnosis and unsuitable patient management procedures [52,58,96,104,250,293,294,295,296]. Therefore, this is more often an issue of the performance of the detection tool (sensitivity and specificity) rather than the contribution of other pathogens to disease severity. Unfortunately, the usefulness of systematic and exhaustive diagnosis in the management of pregnant women (e.g., education and treatment) according to the sequence coinfections, including congenital and arboviral viruses, is already being questioned in some studies [58,104,250,296]. Drawing up a list of potential etiological agents to screen is the next challenge, during which seroprevalence studies need to be considered by firstly focusing on some urban areas associated with particular socioeconomic patterns and sex behaviors in arbovirus endemic regions [52,58,104,250,296]. In light of this review, the maternal coexposure to the latent virus showing urogenital tropism in human organisms, such as HSV-2, HHV-6, EBV, B19V, and HPV with ZIKV, needs to be taken into account. Instead of determining a unique etiology of diseases, the importance of co-exposition in the evolution of pathogenesis needs more scientific and social attention to answer ZIKV infection-related questions. CZS calls for a new diagnostic concept open to multiple etiological definitions [297]. If so, toward which diagnosis tools should the scientific community turn to overcome technical limitations? As mentioned above, coinfections often result in limited sensibility and sensitivity (false negative and positive results). Apart from their etiological cause, the presence of a second pathogen can influence the viral load and limit virus isolation and culture in suitable cell models. In view of the various levels of contribution of pathogens to disease outcomes, the design of tools allowing prioritization may help management, provided data are used properly and can be extrapolated easily. Mixed infections generate heterologous responses of the host immunity and metabolism. Therefore, both laboratory and field work will be necessary to continue developing cutting-edge diagnosis tools by, in particular, thinking about new molecular determinants (e.g., genomes, proteins, and metabolites, their conjugated detection, and the nature of the sample (e.g., blood, urine, saliva). HLV participation in CZS also highlights the need for an in-depth discussion of the management of expectant mothers.

### 4.2. Treatment and Management

The re-emergence of ZIKV, despite the development of congenital diseases still being subject to much controversy in the literature, should lead to regular updates of the relevant pregnancy guidelines. Regarding the hypothesis that the evolution of ZIKV pathogenicity results from opportunistic interplay with HLV rather than new acquisitions, this may result in dramatic recasting of the current CZS management strategies through first targeting latent viruses. In other words, the control of and cure for latency infections may prevent, protect from, and limit the emergence of CZS and other types of ZIKV pathogenicities. In contrast to acute infectious diseases, the measures to eradicate the persistent viruses themselves are still facing challenges.

Apart from improving clinical symptoms and limiting the frequency of recurrence, antiviral strategies have to avoid maintaining virus latency in hosts by continuously focusing on its evolution (RNA genome), its growth in diversity (variant selection), and/or possible superinfection-induced reactivation.

Recent advances in CRISPR/Cas9 genome editing allow for the proposal of cutting-edge strategies for selectively targeting latent viruses by deleting or mutating specific latent virus DNA regions [298]. For example, the use of pharmacological inhibitors of histone demethylase to promote the epigenetic suppression of viral genomes shows a “locking in latency” system for HSV [299,300]. In the same way, certain drugs may allow to release latent viruses and to promote lytic infections as a “lytic therapy” system, as opposed to the former systems [301]. Some of the available antiviral drugs and adjuvants have already shown promise in lytic virus reactivation [142,143]. Using bacteriophage to block reactivation or to promote lysogenic is proposed via a vaccine concept, Acyclovir R430 [302]. Besides targeting latent viruses, treatment combinations should also be proposed, such as antiviral molecular drugs for ZIKV and an immunomodulatory compound for latent viruses. Nevertheless, the heterologous response features of coinfections bring some limitations and risks of inefficiency or exacerbating the immune system, as per common cytokine storms [49]. Such an immune host response is currently at the heart of COVID-19 pathogenesis, for which a preventive approach is a public health priority. This also raises the challenge of vaccine design for ZIKV with co-circulation, co-exposition, and coinfection risks in some biodiversity areas to achieve true efficacy.

### 4.3. Vaccine

The development of a ZIKV vaccine remains a priority. The DENV/ZIKV cross-reactivity preventive effect (the idea that prior DENV infection could provide a protective effect in endemic arbovirus areas) is one of the possible research avenues. However, the risk of DENV antibody production driving antibody-dependent enhancement (ADE) of ZIKV replication has also been characterized, without forgetting the uncertain efficacy of a vaccine to prevent multi-exposition risk, such as DENV, ZIKV, and CHIKV. Therefore, the challenge is to design a vaccine by avoiding (limiting) cross-reactive epitopes, attrition, and depletion or displacement of memory CD8 T cells [49]. Determining the outcome of vaccinations in heterologous infections and ensuring the safety and efficiency are key requirements for the development of any vaccine.

The development of a vaccine based on the concept of the measles vaccine, i.e., providing protection against other unrelated infections, is ongoing [303,304]. In general, the administering of two potential vaccines for endemic areas are proposed: one administered during the outbreak with a vaccination program targeting women of reproductive age to prevent in utero infection, and the other during an inte-epidemic period with a broad-based vaccination campaign targeting the general population to establish population immunity. By contrast, the implementation of a vaccine/treatment to pregnant women remains a dilemma in monoinfections, as well as in mixed infections. In fact, rather than questioning, here, the complexity, necessity, and efficiency of a vaccine design, the complexity of the ZIKV biological system and, especially, of the syndemics process, encourages the preparation of a global and multidisciplinary plan that takes into consideration the risk determinants in CZS emergence and epicenter generation.

### 4.4. Prevention and Primary Healthcare

Despite the severity in congenital outcomes, pregnant women are not differently affected by ZIKV compared to the general population. The non-specific, or worse, the asymptomatic forms in pregnancy highlight the need for greater efforts to implement preventive strategies. Besides the vaccine program, other priorities should be resolved beforehand, including primary healthcare and improving the sanitary conditions of populations. From the perspective of congenital and arbovirus protective advice, the guidelines and education (e.g., sex and hygiene) need to be rethought in terms of the nature and opportunities (frequency) of coinfections in association with congenital risks. The overlapping between the risks area to contract diseases and the CZS emergence epicenter defines an emergency zone. In Rio de Janeiro, 41% of pregnant women infected with arboviruses and other infections lived in urban slums, mostly in Niterói; surprisingly, no coinfections with arboviruses were detected [305].

Environmental factors (e.g., climate), housing conditions, nutrition, and healthcare access as well as behavior regarding the risk of contracting infectious diseases (e.g., sexual activity or outdoor activities (bites from a mosquito), have a combined impact on immune status, without forgetting the genetic background of a person, such as their syndemic image [84,119,120,306].

As a consequence, the acquisition sequence of a mixed infection and its effects on health are unique to each person. All of these factors contribute to a mosaic of response profiles to ZIKV infection in pregnancy between mothers (e.g., symptomatic or asymptomatic, phenotypes, disease progression, and later outcome in infants). While the emergence of ZIKV is a global concern, CZS management still remains singular to individual mothers.

### 4.5. Environmental Surveillance

The climate impacts biological systems [83]; it has differential effects on the surrounding landscapes and shapes urban and rural areas. It also influences the life cycle of pathogens (e.g., replication and temperature), vector ecology (e.g., food behavior and habitats), and the health of different populations (e.g., vulnerability, hygiene, and food access) [122].

In a same region, the climate is included in the definition of the immune profiles of the local populations, as well as in the moving of coinfection opportunities of ZIKV during pregnancy. Therefore, environmental change may be an important sensitive marker of the emergence of infectious disease outbreaks, and also of the risk of coinfections, highlighting the necessity of more monitoring systems linking such environmental changes with the emergence of infectious diseases (EID), and of rethinking, as of now, the concept for future studies.

### 4.6. New Experimental Study Designs for Better Understanding and Preparing for the Future

As described above, several strategies are used to counteract the antiviral host cell process that promotes viral persistence and survival by suitably facilitating reactivation. Moreover, certain common threads of the latency mechanisms appear among human viruses, which is proof of the long-term evolutionary relationships that exist between host organisms and this new viral invader.

The characterization of the common mechanisms involved in latent coinfections with ZIKV is key to developing suitable prevention strategies for prior and/or during pregnancy, thus limiting the risk of CZS. For this, models need to be designed to reproduce co-viral infections and environments.

Thus far, mathematical models of viral coinfections have already been developed for HIV/HCV coinfection, allowing for the determination of risk factors (e.g., human activity), to evaluate antiviral drug combinations, predict disease progression and, possibly, to elucidate drug resistance as a result of viral genome coevolution [49]. Even if this offers wide opportunities for concepts to study several determinants and interactions, a better understanding of the molecular and cellular processes controlling viral latency in ZIKV infection cannot be accurately addressed using these mathematical models.

Several types of persistent infections can already be reproduced in cell culture systems depending on viral latent forms (e.g., chronic focal infections, chronic diffuse infections, and true latent infections) [131]. Interestingly, an induced pluripotent stem cell-derived (iPSC-derived) myeloid lineage model of herpes virus latency has been proposed as a potential support for studying ZIKV [307].

Nevertheless, the establishment and maintenance of latency depends on tissue microenvironments and often cannot be recapitulated in cell culture. As a consequence, complex tissue culture models are presumably required to replicate some aspects of viral latency. For example, HSV1 latency can be modeled in primary rat neurons treated with nerve growth factor (NGF), which maintains progenitor neuronal cell survival and prevents viral lytic cycle gene expression [308]. This could be suitable in a ZIKV/HSV coinfection model for understanding the observed neurotropism and neuropathogenicity in adults as well as in fetal development [142,143].

Following this, the contribution of coinfections to the outcome of diseases as compared to monoinfections needs support from studies using physiological (animal) models. Such physiological systems should help us to better understand the enigmatic outcome of ZIKV infection in twin pregnancies [309].

In addition to the confounding clinical signs, multiple misdiagnosis opportunities, unsuitable management, and cures with others viral congenital pathogens, the experimental evidence takes us even further away from claiming a causal link between ZIKV and microcephaly, and wrongly so.

In addition to genetic evolution studies, omics tools (e.g., transcriptomics and proteomics) may allow to deepen our understanding of the effects of coinfections on host physiological, endocrine, and immune response changes. Metabolomic and lipidomics will occupy a major place in the future diagnostic tools for investigating arboviruses (e.g., ZIKV) and could be the next field of ZIKV/latent virus coinfection research (e.g., physiopathology studies, diagnostics, and treatment) [310].

These last challenges call upon to firstly review of the perspectives in terms of biodiversity awareness and respect.

## 5. General Conclusions and Perspectives

The Brazilian ZIKV outbreak marks a strong comeback of vector-borne pathogens to the forefront of the international arena. In summary, CZS mirrors a long virus evolution built along three major periods with potential overlap: (1) Historically, the definition of ZIKV was glued to the classical definition of arbovirus as a non-threatening infection, but with the potential for emergence (e.g., in Micronesia and, specifically, Yap) (Figure 1). The ZIKV life cycle is balanced between anthropophilic *Aedes aegypti* (the vector) and opportunistic urban humans (the host), allowing occasional meetings with other vertebrate pathogens, such as persistent and/or congenital viruses whose genomes are mostly DNA (Figure 3 and Table 1). (2) The current state of globalization generates vector/human promiscuity, resulting in the intensification and increase in the frequency of ZIKV human infections. This context leads to humans being a suitable host for ZIKV, with adaptations in the viral replication, the immune dialogue, and the pathogenicity outcome (Figure 4). The heterogeneous behavior of populations provides a mosaic of opportunities for parallel evolution (coevolution) with latent/congenital viruses in humans, from which it obtains new properties, e.g., persistent and possible sexual transmission and congenital tropism. Thus, could we be speaking of a human reservoir and a true congenital pathogen? (3) Humans are already a reservoir of congenital viruses, meaning mostly persistent viruses with the potential for reactivation. Latent viruses show uniqueness in a persistent process in which the viral genome remains dormant in cells throughout the host lifetime.

Their reactivation and subsequent triggering of lytic replication leads to large amounts of viral progeny that can be transmitted to new hosts, such as from mother to fetus in pregnancy, without new external reinfections. The physiological changes in pregnancy may trigger reactivation of latent viruses as well as infection by another virus. ZIKV infection in pregnancy provides a dual effect: both reactivation and co-exposition opportunities for latent viruses (Figure 5). In turn, the viral lytic cycle mechanism causes biochemical, cellular, immune, and physiologic process disorders in women, which results in a fatal outcome for fetal development, overall, in a ZIKV (co)infected mother context. Microcephaly is commonly the clinical key between congenital pathogens.

Ubiquitous in human compartments, the persistent viruses described here result in the acquisition of a large spectrum of Congenital ZIKV Syndrome, clinical signs, and disease progressions, all shaped by host response.

As a consequence, should the next direction of the evolution of ZIKV be to determine a latent virus image with inter-human dissemination and a strict life cycle in vertebrates (e.g., transmission, replication, and persistence) with the expansion of genome diversity? If so, what about the upcoming (future) pathogenesis phenotypes? Where, when, and how? Perhaps a better answer refers to the past: Where did ZIKV come from?

ZIKV is thought to be a zoonotic virus from an as-yet-uncertain animal reservoir (zoonosis). Studies on coinfections in wildlife need to be performed and questions must be asked about their viral latency contribution to the emergence of viral diseases.

The growing complexity of systems is a key pattern for viral adaptation, and, thus, is unpredictable, but against which it is possible to prepare, protect, limit, and control effects.

To this end, “One Health” research needs to be reinforced from the epidemiological, clinical, and experimental perspectives. Apart from the matter of arboviruses, scientific efforts are also helpful for fighting other currently emerging fatal viruses, such as fever hemorrhagic Ebola virus in Africa and COVID-19 worldwide. The global increase in COVID-19 incidence with atypical clinical manifestations and even fatalities, though worse in extent, call to mind the prior ZIKV scenario in Brazil. Then, what is the impact of latent viruses on the pathogenicity of COVID-19 and on its global and continuous emergence?

## Figures and Tables

**Table 1:** Human latent virus (HLV) general characterization: classification, genome, viral cycle, pathogenicity, host response, transmission, pathogenesis, and epidemiology (AT (aero-pharyngeal transmission), VT (Vertical Transmission), IC (Intia Contact)).

Items	Species	HSV-2	HHV-6	EBV	PB19	HPV
**Name**	**Family**	Herpesviridae			Parvoviridae	Papillomaviridae
	**Subfamily**	Alphaherpesvirinae	Betaherpesvirinae	Gammaherpesvirinae	Parvovirinae	-
	**Genre**	Simplex virus	Simplex virus	Lymphocryptovirus	Erythroparvovirus	Alphapapillomavirus
**Genome**	**Nature**	dsDNA	dsDNA	dsDNA	ssDNA	dsDNA
	**Form**	Linear	Linear	Linear	Circular	Circular
	**Size**	120-180 kb	140-240kb	180 kb	4-6 kb	8 kb
**Viral cycle**	**Cell types**	Epithelial	TCD4 cell	Memory B cell	Erythroid precursor	Mucosa epithelium
	**Transcription**	Nucleus	Nucleus	Nucleus	Nucleus	Nucleus
	**Replication kinetic**	Short (hours)	Long (days)	Latency dominance	Short (hours)	Short (hours)
**Pathogenicity**	**Tissue tropism**	Genital tract (GT)	Ubiquitous	Ubiquitous	Bone marrow, fetal liver	Mouth, thorax, GT
	**Receptor**	TAM family	CD46?	PDL-1/PD-1	Globoside, α5β1 integrin	α6β4 integrin
	**Cell effects**	Pro-inflammation, apoptosis	Immunomodulation, apoptosis	Tumorgenesis (B-cell immortalization)	Erythropoiesis blockage (red blood cell precursors death)	Malignancy (abnormal cell growth and differentiation)
**Host response**	**Immune response**	Inflammation (IL-1b, IL-8), inflammasome activation (NLRP3)	Cell specificity immunomodulation	Anti-apoptosis, antiviral immune control, genome instability	Inflammation (IL-2, IL-6,) and anti-apoptosis (NF-κB)	Th2 dominance and prolonged response (IL-5, IL-10, IL-17A)
**Transmission**	**Mode**	IC, VT, perinatal	AT, saliva, VT	AT, saliva	AT, saliva	IC
	**Host behavior risk**	Risky sexual behaviors	Close human contact	Close human contact	Hematological, immunological state	Risky sexual behaviors
**Pathogenesis**	**Primo-infection**	Genital lesions (warts), 24% symptomatic	HHV-6B: Roseola infantum or 6th disease	Mononucleosis-like syndrome	Slapped cheek syndrome or 5th disease	Genital warts resolving within 2 years
	**Progression**	Latency (20%–50%)	Latency (70%-100%)	Nasopharyngeal carci- noma, lymphoma,	Cardio-, hepato-, neuro-pathies	Cervical cancer 70%: HPV16/18 subtypes
	**Vulnerable person**	Reproductive life woman	Toddlers (2–3 years of age)	Immuno-depressive person	Immunologic & hematologic disorders	Reproductive life woman, immuno-depressive person
**Epidemiology**	**Key point**	(+) 11.3% of global seroprevalence	(+) 90% of the toddlers	(+) 90% of the global population	(+) mostly of the school ages children	100% of cervical cancer origin

**Table 2:** Viral latency features: sites of latency, viral forms, mechanisms of establishment, and maintenance.

Items	Species	HSV-2	HHV-6	EBV	PB19	HPV
**Non-infectious reactivation**	**Factors**	Environnemental stressors (UV exposition, hypoxia, trauma, pain), hormonal treatment, immunosuppression state, or «spontaneous»	Pregnancy?	Environnemental stressors (UV exposition, hypoxia), hormonal treatment, immunosuppression state	Oxygen, stress, Pregnancy	Environmental stressors (UV exposition, hypoxia), smoking, hormonal treatment, pregnancy, genital wart
**Pathogen-induced reactivation and consequences**	**Virus triggers**	HIV, HPV	EBV, CMV	HIV	Unknown	EBV, HSV-2, HHV-6, PB19
	**Consequences**	HIV sexual transmission, HPV-related cervical cancer	EBV and CMV antibodies production, CMV lymphopenia aggravation	B-cell immortalization, anti-apoptotic signalization, tumorgenesis	-	HPV genomic instability (EBV), HPV oncogenesis (HSV-2), HPV clearance inhibition (HHV-6)
**Pregnancy induced reactivation (woman pre-conceptual)**	**Context**	Recurrent genital herpes	HHV6-A variant	35% of reactivation related to HIV coinfection	13–20 weeks pregnancy (fetal oxygen demand)	Genital warts (mostly asymptomatic)
	**Consequences**	ST and VT	1% of VT	?	VT	VT and PT
	**Pregnancy outcome**	Abortion, low birth weight, premature delivery	Infertility, miscarriage, embryogenesis affect	Placental cells changes, Th2 predominance at placental interface	Hypoxia-induced inflammation and fetal abnormal development	Infertility, abortion, choriocarcinoma

**Table 3:** Human latent virus (HLV) reactivation, ZIKV infection, and pregnancy: factors for reactivation, pathogen-induced reactivation, and pregnancy-induced reactivation.

Items	Species	HSV-2	HHV-6	EBV	PB19	HPV
**Latency sites**	**Cell types**	Neuronal cells	CD34+HSCs	Memory B cells	Erythroid progenitor cells	Epithelial cells (basal stem cells)
	**Tissues tropism**	Sensory nerves	Lymphoid organs (e.g. spleen), lymph nodes)	Salivary glands, lymphoid organs	Unspecific tissues (e.g. skin, liver, synovial membrane)	Skin (epidermis), Genital tract (cervix), oral cavity (salivary glands, tonsillar crypts)
**Latency forms**	**Viral DNA form**	Circular episome	Proviral form	Circular, episome	Proviral form	Circular episome
	**Cell DNA link**	Free	Integrated	Linked	Integrated	Linked
	**Localization**	Nucleus	Nucleus	Nucleus	Nucleus	Nucleus
**Latency establishment**	**Viral DNA-host DNA relation**	HSV-2 DNA “chromatin form” and independent	Viral genome as a integral part of host chromosome	Host DNA binding via specific structurally region	Viral genome as a integral part of host chromosome	Host DNA binding via specific structurally region
	**Host cell cycle**	Only in non-dividing cells	Synchronic duplication (S phase)	Synchronic duplication (S phase)	S and G2/M phases arrest	S phase quiescent
	**Viral genome keys**		Telomeric junction area integration	Chromosomally tethered virus	Palindromic repeat sequences	Chromosomally tethered virus
**Latency maintain**	**Principe**	Limited viral transcription	Limited viral transcription	Survival immortalization infected B cells	Down-regulated erythroid cell cycle	Non-cyclic cells growth and tumorgenesis
	**Regulation**	Epigenetic process	Epigenetic process	Epigenetic process	DNA replicative machinery using and DDR disturbing	DNA replicative machinery using and DDR disturbing
	**Key factors**	LATs, ICP10, miRNA	H6-LTs, ORFs, U94, miRNA-U86	EBNA1, OBPs, LMPs, EBNA-2, EBNA-LP, miRNA	Hypoxia, NS1, EPO/EPO rcp.	HPV6/7 activators, HPV2 replication

## Data Availability

Not applicable.
